# SARS-CoV-2 infection in patients with autoimmune hepatitis

**DOI:** 10.1016/j.jhep.2021.01.021

**Published:** 2021-06

**Authors:** Thomas Marjot, Gustav Buescher, Marcial Sebode, Eleanor Barnes, A. Sidney Barritt, Matthew J. Armstrong, Luke Baldelli, James Kennedy, Carolyn Mercer, Ann-Kathrin Ozga, Christian Casar, Christoph Schramm, contributing Members and Collaborators of ERN RARE-LIVER/COVID-Hep/SECURE-Cirrhosis, Andrew M. Moon, Gwilym J. Webb, Ansgar W. Lohse

**Affiliations:** 1 Oxford Liver Unit, Translational Gastroenterology Unit, Oxford University Hospitals NHS Foundation Trust, University of Oxford, Oxford, UK; 2 Department of Medicine, University Medical Centre Hamburg-Eppendorf, Hamburg, Germany; 3 European Reference Network on Hepatological Diseases (ERN RARE-LIVER), Germany; 4 Division of Gastroenterology and Hepatology, University of North Carolina, Chapel Hill, North Carolina, USA; 5 Liver Unit, Queen Elizabeth Hospital Birmingham, Birmingham, UK; 6 Department of Medicine, University of North Carolina, Chapel Hill, North Carolina, USA; 7 Institute of Medical Biometry and Epidemiology, University Medical Center Hamburg-Eppendorf, Hamburg, Germany; 8 Martin Zeitz Centre for Rare Diseases, University Medical Centre Hamburg-Eppendorf, Hamburg, Germany; 9 Cambridge Liver Unit, Addenbrooke’s Hospital, Cambridge University Hospitals, Cambridge, UK

**Keywords:** autoimmune hepatitis, SARS-CoV-2, COVID-19, immunosuppression, coronavirus, liver disease

## Abstract

**Background & Aims:**

Severe acute respiratory syndrome coronavirus 2 (SARS-CoV-2) and coronavirus disease 2019 (COVID-19) continues to have a devastating impact across the globe. However, little is known about the disease course in patients with autoimmune hepatitis (AIH).

**Methods:**

Data for patients with AIH and SARS-CoV-2 infection were combined from 3 international reporting registries and outcomes were compared to those in patients with chronic liver disease of other aetiology (non-AIH CLD) and to patients without liver disease (non-CLD).

**Results:**

Between 25^th^ March and 24^th^ October 2020, data were collected for 932 patients with CLD and SARS-CoV-2 infection including 70 with autoimmune hepatitis (AIH). Fifty-eight (83%) patients with AIH were taking ≥1 immunosuppressive drug. There were no differences in rates of major outcomes between patients with AIH and non-AIH CLD, including hospitalization (76% *vs.* 85%; *p =* 0.06), intensive care unit admission (29% *vs.* 23%; *p =* 0.240), and death (23% *vs.* 20%; *p =* 0.643). Factors associated with death within the AIH cohort included age (odds ratio [OR] 2.16/10 years; 1.07–3.81), and Child-Pugh class B (OR 42.48; 4.40–409.53), and C (OR 69.30; 2.83–1694.50) cirrhosis, but not use of immunosuppression. Propensity score matched (PSM) analysis comparing patients with AIH with non-AIH CLD demonstrated no increased risk of adverse outcomes including death (+3.2%; -9.2%–15.7%). PSM analysis of patients with AIH *vs*. non-CLD (n = 769) demonstrated increased risk of hospitalization with AIH (+18.4%; 5.6–31.2%), but equivalent risk of all other outcomes including death (+3.2%; -9.1%–15.6%).

**Conclusion:**

Patients with AIH were not at increased risk of adverse outcomes despite immunosuppressive treatment compared to other causes of CLD and to matched cases without liver disease.

**Lay summary:**

Little is known about the outcomes of COVID-19 in patients with autoimmune hepatitis (AIH), a rare chronic inflammatory liver disease. This study combines data from 3 large registries to describe the course of COVID-19 in this patient group. We show that AIH patients do not appear to have an increased risk of death from COVID-19 compared to patients with other forms of liver disease and compared to patients without liver disease, despite the use of medications which suppress the immune system.

## Highlights

• This is the largest cohort of patients with autoimmune hepatitis and laboratory proven SARS-COV-2 infection reported to date.
• There were no differences in rates of major adverse COVID-19 outcomes between patients with AIH and those with other liver diseases.
• Patients with AIH had higher rates of hospitalization than patients without liver disease, but no increased risk of ICU admission or death.
• Independent risk factors for death in AIH patients were age and baseline liver disease severity, but not the use of immunosuppression.

## Graphical abstract

**Figure:**
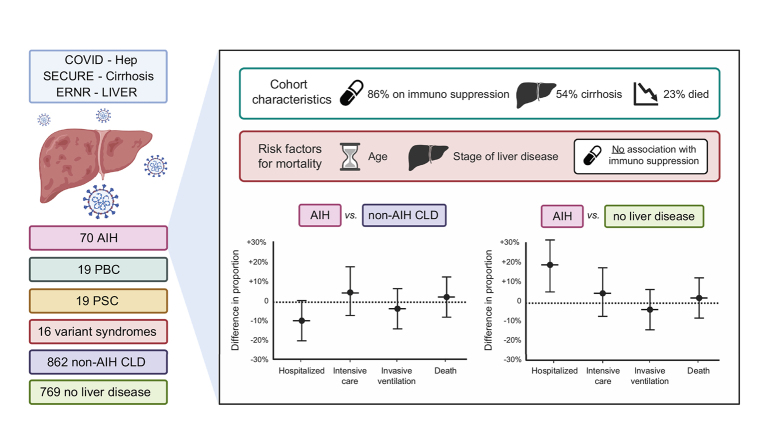

## Introduction

Severe acute respiratory syndrome coronavirus 2 (SARS-CoV-2) infection and resultant coronavirus disease 2019 (COVID-19) continues to have a devastating impact across the globe.^1^ Since the onset of the pandemic, the scientific and clinical community have strived to understand the contributions of specific disease phenotypes to SARS-COV-2 susceptibility and subsequent adverse outcomes.^2^ Recently, large international and multicentre cohorts have shown baseline liver disease severity and alcohol-related liver disease (ALD) to be independently associated with COVID-19 mortality, with decompensated cirrhosis representing a particularly high-risk group.[3], [4], [5], [6] In contrast, several studies have demonstrated no significant increased risk of critical COVID-19 in patients with previous liver transplantation despite high rates of immunosuppression.[7], [8], [9] However, no studies have yet evaluated the disease course and outcomes specifically for patients with autoimmune hepatitis (AIH), a large proportion of which will be on concurrent immunosuppressive agents.

The clinical impact of pre-existing immunosuppression in COVID-19 remains complex and incompletely defined. Observations in inflammatory bowel disease and rheumatological conditions have suggested a more severe disease course in those under maintenance treatment with thiopurines or corticosteroids, respectively.^10^^,^^11^ Furthermore, a multicentre study in Spain demonstrated a higher incidence of SARS-CoV-2 in immunosuppressed liver transplant recipients compared with the general population.^8^ In contrast, dexamethasone now has an established role in the management of hospitalised patients with COVID-19 potentially through modification of the hyperactive immune response, and has been shown to reduce mortality by a third in intubated patients and a fifth in those requiring supplemental oxygen therapy.^12^

Current expert recommendations advocate against the routine modification of immunosuppressive therapy in patients with AIH both before and after SARS-CoV-2 infection.^13^^,^^14^ However, there is little evidence beyond expert consensus and very small cohorts to support these recommendations.^15^^,^^16^ Furthermore, given the resurgence of the virus in many areas of the world, clinicians and policy makers are being forced to carefully risk stratify patients to establish who may benefit most from enhanced physical and social distancing. A detailed understanding of the clinical course of COVID-19 in patients with AIH is therefore urgently required.

The current study represents an international collaborative effort, bringing together data from 3 large-scale reporting registries: The European Association for Study of the Liver (EASL) supported COVID-Hep registry, the American Association for the Study of Liver Diseases (AASLD) supported SECURE-cirrhosis registry and the European Reference Network on Hepatological Diseases (ERN RARE-LIVER). To our knowledge, we describe the epidemiology, presentation, disease course and outcomes of the largest cohort of patients with SARS-CoV-2 infection and AIH and offer statistical comparisons with liver disease of other aetiologies and to a contemporaneous cohort of patients without liver disease who tested positive for SARS-COV-2.

## Patients and methods

### Setting and study design

We combined the data from 3 multinational registries for patients with laboratory confirmed SARS-CoV-2 and AIH collected between March 25^th^ 2020 and 24th October 2020. These registries included the R-LIVER COVID-19 registry (co-ordinated by the ERN RARE-LIVER, with R-LIVER being the general registry of ERN RARE-LIVER), the SECURE-Cirrhosis registry (co-ordinated by University of North Carolina, USA, and supported by AASLD), and the COVID-Hep.net registry (co-ordinated by University of Oxford, UK, and supported by EASL). All 3 registries were widely advertised through the communication channels of multiple endorsing gastroenterology and hepatology societies, direct emails to hepatology providers, and through social media. Submitting clinicians were asked to complete a case report form of clinical data at the end of their patient’s disease course, defined as resolution of clinical signs of COVID-19, discharge from hospital, or death. All 3 registries used an online reporting form which was identical for COVID-Hep and SECURE-cirrhosis, but different for the R-LIVER COVID-19 registry; copies of both data collection tools are available as a supplementary annex. In order to centralise and amalgamate overlapping report form information, case data from the R-LIVER report form was re-entered onto the online COVID-Hep report form via www.COVID-Hep.net.

Whereas the R-LIVER COVID-19 registry collected data exclusively for autoimmune liver disease and other rare liver diseases, COVID-Hep.net and SECURE-Cirrhosis registries also simultaneously collected identical data for patients with laboratory confirmed SARS-CoV-2 and all other aetiologies of chronic liver disease (CLD). The current study includes 745 cases of CLD and SARS-CoV-2 infection, including 42 patients with AIH, which are included in a recently published analysis from the COVID-Hep/SECURE-cirrhosis registry. However, this prior publication did not include any analysis on patients with AIH. In addition, 2 recent publications from Italy partially contained descriptive data from 4 patients included in the registries of this study.^16^^,^^17^ Contributing centres of ERN RARE-LIVER were asked to report the total number of patients with AIH in their institutions and to report monthly even if no cases of SARS-CoV-2 were identified in their patients with AIH.

To provide a comparison cohort of patients without CLD, data were also extracted using the SECURE-cirrhosis/COVID-Hep data collection tool from electronic patient records of consecutive patients testing positive for SARS-CoV-2 over the same time period at Oxford University Hospitals NHS Foundation Trust (OUHFT), an organization of 4 hospitals in and around Oxford in the UK, and from the University of North Carolina Hospitals (UNCH). Positive cases from OUHFT and UNCH were defined as detection of SARS-CoV-2 by reverse transcription PCR (RT-PCR) on nasopharyngeal swabs. Any cases of SARS-CoV-2 infection in patients with pre-existing CLD who were identified from the electronic health records whilst compiling the non-CLD cohort were subsequently incorporated into the CLD cohort. To minimize potential reporting bias, data extraction for the non-CLD cohort was performed by investigators blinded to the clinical characteristics and outcomes reported in patients with CLD. All data for both CLD and non-CLD cohorts were uploaded real-time to the same secure, online, data capture tool.

All submitted report forms for all cohorts were manually reviewed to assess data quality, completeness and inconsistencies and in some instances, submitting clinicians were contacted and asked to provide additional data where appropriate. When combining the R-LIVER COVID-19 registry and SECURE-Cirrhosis/COVID-Hep datasets, possible dual reporting to both registries was identified based on the submitting clinician, centre and matching patient demographic information; duplicate entries were removed.

### Ethical and regulatory approval

The data collected contained no personal health identifiers and both SECURE-cirrhosis and COVID-Hep registries were deemed not to constitute human research by the University of North Carolina Office of Human Research Ethics and the University of Oxford Clinical Trials and Research Governance (CTRG) respectively. Formal local audit approval was sought and received for data acquisition from OUHFT electronic health records (ref: OUH5595). The collection of clinical data by R-LIVER was approved by the local ethics committee (ref: PV5548).

### Participants

**Fig. 1:**
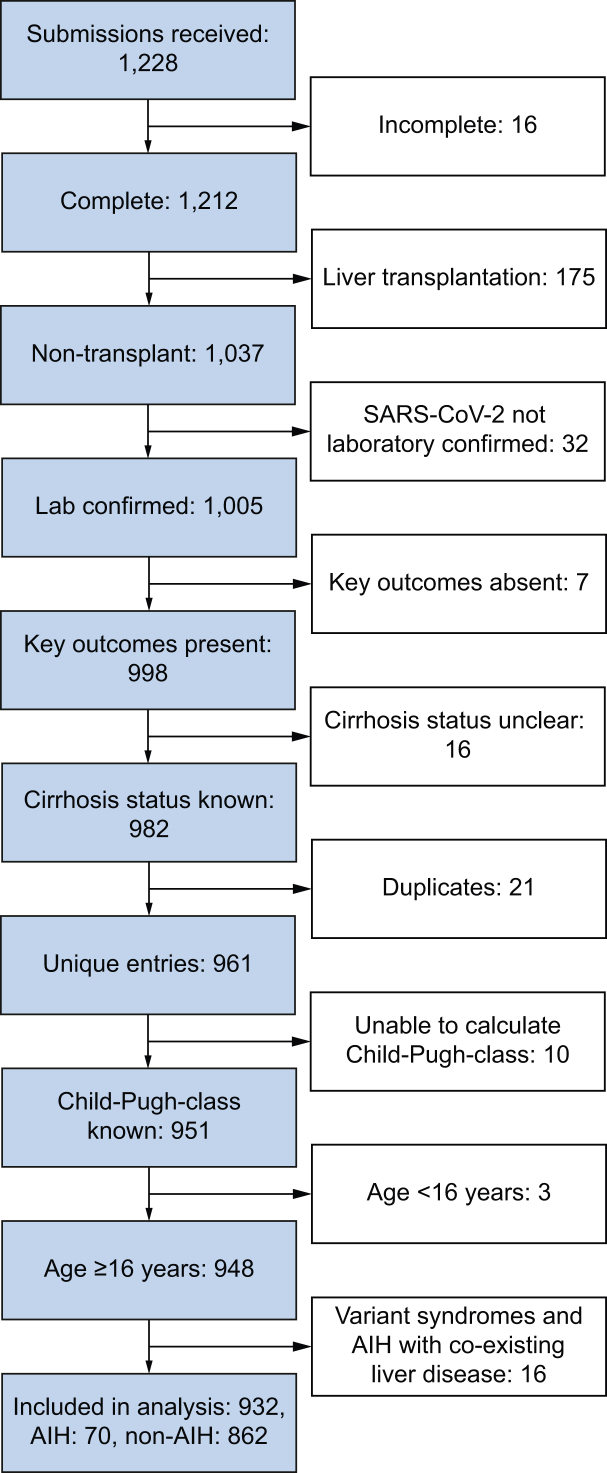
Total combined submissions of patients with CLD and SARS-CoV-2 infection COVID-Hep/SECURE-Cirrhosis, and R-LIVER registries between 25th March and 24th October 2020 and the number included in final analysis after exclusions.

All cases of laboratory-confirmed SARS-CoV-2 infection in patients with CLD without prior liver transplantation, aged >16 years-old (the age for admission under adult services at OUHFT), from any location, and with any symptom profile or disease severity were included in the analysis. Cases were excluded if any of the following conditions were met: SARS-CoV-2 infection was not laboratory-confirmed, the submission was a duplicate, if hospitalization status, cirrhosis status, or mortality outcome was not known or not reported, or if the patient was not aged 16 years or over at the time of a positive SARS-COV-2 diagnosis (Fig. 1). Patients with variant syndromes of primary biliary cholangitis (PBC) and primary sclerosing cholangitis (PSC) (so-called AIH/PBC or AIH/PSC overlap syndromes) and patients with AIH and coexisting liver disease (*e.g.* AIH with alcohol-related liver disease) were excluded from the analysis. This was due to internationally varying diagnostic criteria and difficulty defining the predominant liver disease phenotype of variant syndromes from the data capture tools. Characteristics and outcomes of patients with variant syndromes and AIH with co-existing liver disease are presented in Table S1.

### Variables and definitions

Liver disease stage and aetiology was classified by the reporting clinician. Those with cirrhosis were then further sub-categorized by the reporting clinician according to Child-Pugh class (A, B or C). Throughout this paper the following terminology will be used to define the groups; total CLD cohort (CLD), autoimmune hepatitis cohort excluding variant syndromes and IgG4-related disease (AIH), CLD without AIH (non-AIH CLD), and patients without liver disease (non-CLD).

Obesity was defined as a BMI of >30 kg/m^2^; where data on BMI was unavailable obesity was assumed to be absent. For analysis of ethnicity, only white ethnicity (as the majority classification) as compared to other ethnicities was considered in the analysis. For the non-CLD cohort, where ethnicity was not recorded, white ethnicity was assumed.^18^ A full list of ethnicity classifications can be found in the supplementary annex.

### Statistical methods

Patient factors and outcome are summarized for all cohorts by occurrence of mortality using standard summary statistics (number of events and percentage for binary and median and interquartile range for continuous measures). Univariable analysis of mortality by patient characteristics was performed using logistic regression. Multivariable comparisons of factors associated with death within cohorts were assessed using logistic regression. Only patients with data available for each reported data point (with the exception of assumed values for obesity and ethnicity, as explained above) were used in multivariable analyses. For sensitivity analysis, models were repeated with backwards stepwise selection as described. Fisher’s exact test was used to compare proportions between 2 populations. Exact (Clopper-Pearson) binomial confidence intervals were calculated when describing proportions. The absence of accurate data on the duration from positive laboratory COVID-19 diagnosis to death in the COVID-Hep/SECURE cirrhosis registries prevented a time-dependent analysis (*e.g.* Cox regression and Kaplan-Meier curves). Nominal statistical significance was adopted at a 2-sided 5% level.

To evaluate the effect of the AIH on the COVID-19 disease course we compared major outcomes in patients with AIH to both non-AIH CLD and non-CLD cohorts using propensity score 1:2 matched samples via a nearest neighbour approach (individuals matched according to similar propensity score).^19^ Covariables included in the propensity score model were selected based on their known associations with severe COVID-19,^2^^,^^20^ whilst aiming to provide matched variance ratios of between 0.5–2.0. In AIH *vs*. non-AIH CLD, the variables included were age in years, interactions with age, sex, and baseline liver disease severity (CLD without cirrhosis, Child-Pugh class A, B, or C). Covariables included in the propensity score matched model of AIH *vs.* non-CLD were age, interactions with age, sex, hypertension, chronic obstructive pulmonary disease (COPD), heart disease and diabetes. Propensity score matched analysis was performed using the *teffects* function in Stata. The average treatment effect on the treated (ATET) was calculated with robust Abadie-Imbens standard errors.^19^ All statistical analyses were conducted using Stata v15.1 (College Station, TX). Similar statistical methods have recently been used to evaluate outcomes in the CLD and liver transplantation cohort from the COVID-Hep and SECURE-Cirrhosis registries.^3^^,^^7^

## Results

### Chronic liver disease and AIH cohort

Between 25th March and 24th October 2020 there were a total of 1,228 combined case submissions to SECURE-cirrhosis/COVID-Hep and R-LIVER COVID-19 registries. After exclusions, 932 patients with CLD remained (Fig. 1) from 35 countries including 70 with AIH (n = 70) (Table 1). Of note, data on 2 paediatric patients with AIH were submitted to the registry but were excluded from the analysis; 1 female aged 5 and 1 male aged 10, both of whom had a mild disease course and were not hospitalized. A total of 862 non-AIH CLD were included (Table 2). The major aetiologies within the non-AIH CLD cohort included 362 patients with non-alcoholic liver disease (42%), 233 with alcohol-related liver disease (ALD; 27%), 128 with chronic HCV infection (15%) and 121 with chronic HBV infection (14%). The non-AIH CLD cohort also included 19 patients with PSC (2%) and 19 with PBC (2%). Rates of major co-morbidities are presented in Table 2; the number of patients in the CLD cohort with unknown BMI who were assumed to be non-obese for the analysis was 116/932 (12%).

**Table 1:** Characteristics of AIH cohort and factors associated with death following SARS-CoV-2 infection.

	AIH cohort (n = 70)	Survived (n = 54)	Died (n = 16)	Univariable analysis		Multivariable analysis		Stepwise selection	
	Median or n (IQR/%)	Median or n (IQR/%)	Median or n (IQR/%)	OR (95% CI)	*p* value	OR (95% CI)	*p* value	OR (95% CI)	*p* value
Demographics									
Age (per 10-years)	55 (44-71)	55 (38-68)	57 (47-75)	1.18 (0.86–1.63)	0.298	2.01 (1.07–3.81)	**0.031**	1.75 (1.10–2.80)	**0.018**
Sex (male)	21 (30%)	17 (31%)	4 (25%)	0.73 (0.20-2.58)	0.620	0.35 (0.06–2.16)	0.257	0.33 (0.06–1.80)	0.199
Ethnicity (white)	33 (47%)	24 (44%)	9 (56%)	1.61 (0.52–4.95)	0.408	0.83 (0.19–3.73)	0.813		
Liver disease severity									
CLD without cirrhosis	32 (46%)	29 (54%)	3 (19%)	1.00 (REF)		1.00 (REF)		1.00 (REF)	
Child-Pugh A	17 (24%)	15 (28%)	2 (13%)	1.29 (0.19–8.57)	0.793	2.91 (0.23–37.51)	0.412	3.14 (0.36–27.46)	0.301
Child-Pugh B	13 (19%)	6 (11%)	7 (44%)	11.28 (2.25–56.59)	**0.003**	42.48 (4.41–409.53)	**0.001**	32.93 (4.20–258.07)	**0.001**
Child-Pugh C	8 (11%)	4 (7%)	4 (25%)	9.67 (1.56–60.01)	**0.015**	69.30 (2.83–1,694.50)	**0.009**	62.00 (4.68–821.987)	**0.002**
Co-factors									
Any immunosuppression	58 (83%)	47 (87%)	11 (69%)	0.33 (0.09–1.23)	0.098	0.79 (0.10–6.25)	0.822		
Heart disease	9 (13%)	6 (11%)	3 (19%)	1.85 (0.41–8.40)	0.428	0.90 (0.09–8.51)	0.925		
Diabetes mellitus	11 (16%)	10 (19%)	1 (6%)	0.29 (0.03–2.49)	0.261	0.22 (0.01–5.03)	0.344	0.13 (0.01–2.76)	0.191
COPD	3 (4%)	2 (4%)	1 (6%)	1.73 (0.15–20.46)	0.662	0.93 (0.04–21.03)	0.966		
Hypertension	19 (27%)	15 (28%)	4 (25%)	0.87 (0.24–3.11)	0.826	0.35 (0.04–2.77)	0.317		
Baseline laboratory values									
Bilirubin (mg/dl)	1.0 (0.6-2.1)	0.7(0.6-1.5)	2.1 (1.2-3.7)	1.12 (0.95–1.34)	0.175				
Serum albumin (g/dl)	3.8 (3.0-4.2)	4.0 (3.4-4.2)	3.2 (2.7-3.6)	0.86 (0.77–0.96)	**0.009**				
Prothrombin time (s)	13 (12-17)	13 (11-15)	15 (14-22)	1.15 (0.99–1.34)	0.075				

Patient characteristics of AIH patients with laboratory-confirmed SARS-CoV-2 infection. Univariable associations with death and associated *p* values assessed by logistic regression. Multivariable analysis for association with death performed using logistic regression including all variables apart from laboratory values which form part of the Child-Pugh classification. CLD, chronic liver disease; COPD, chronic obstructive pulmonary disease; OR, odds ratio.

**Table 2:** Characteristics of total CLD cohort and association of liver disease aetiology including AIH with death.

	Total CLD (n = 932)	Survived (n = 742)	Died (n = 190)	Univariable analysis		Multivariable analysis		Stepwise selection	
	Median or n (IQR/%)	Median or n (IQR/%)	Median or n (IQR/%)	OR (95% CI)	*p* value	OR (95% CI)	*p* value	OR (95% CI)	*p* value
Demographics									
Age (per 10-years)	59 (48-68)	57 (46-67)	63 (53-73)	1.03 (1.02–1.04)	**<0.001**	1.27 (1.09–1.50)	**0.003**	1.30 (1.12–1.52)	**0.001**
Sex (male)	583 (67%)	463 (62%)	120 (63%)	1.03 (0.74–1.44)	0.847	0.77 (0.51–1.17)	0.221		
Ethnicity (white)	442 (47%)	320 (43%)	122 (64%)	2.37 (1.70–3.29)	**<0.001**	1.37 (0.92–2.04)	0.124	1.34 (0.91–1.99)	0.137
Liver disease severity									
CLD without cirrhosis	423 (45%)	394 (53%)	29 (15%)	1.00 (REF)		1.00 (REF)			
Child-Pugh A	231 (25%)	188 (25%)	43 (23%)	0.86 (0.59–1.26)	0.441	2.18 (1.24–3.84)	**0.007**	2.21 (1.29–3.78)	**0.004**
Child-Pugh B	163 (18%)	105 (14%)	58 (31%)	2.67 (1.84–3.86)	**<0.001**	4.79 (2.72–8.45)	**<0.001**	5.04 (2.92–8.73)	**<0.001**
Child-Pugh C	115 (12%)	55 (7%)	60 (32%)	5.77 (3.82–8.70)	**<0.001**	12.41 (6.73–22.88)	**<0.001**	12.43 (6.89–22.43)	**<0.001**
Aetiology									
AIH	70 (8%)	54 (7%)	16 (8%)	1.17 (0.65–2.09)	0.594	1.87 (0.81–4.34)	0.145	1.87 (0.89–3.90)	0.097
NAFLD	362 (39%)	308 (42%)	54 (28%)	0.56 (0.40–0.79)	**0.001**	0.98 (0.56–1.71)	0.946		
ALD	233 (25%)	150 (20%)	83 (44%)	3.06 (2.18–4.29)	**<0.001**	1.79 (1.06–3.01)	**0.029**	1.66 (1.09–2.55)	**0.018**
HCV	128 (14%)	98 (13%)	30 (16%)	1.23 (0.79–1.92)	0.357	1.05 (0.59–1.88)	0.87		
HBV	121 (13%)	108 (15%)	13 (7%)	0.43 (0.24–0.78)	**0.006**	0.96 (0.45–2.07)	0.925		
Co-factors									
Smoker	67 (7%)	55 (7%)	12 (6%)	0.84 (0.44–1.61)	0.602	0.53 (0.25–1.14)	0.106	0.51 (0.24–1.09)	0.081
Obesity	248 (27%)	197 (27%)	51 (27%)	1.02 (0.71–1.45)	0.935	1.07 (0.69–1.65)	0.767		
Heart disease	165 (18%)	114 (15%)	51 (27%)	2.02 (1.39–2.95)	**<0.001**	1.41 (0.88–2.26)	0.151	1.52 (0.96–2.40)	0.071
Diabetes	339 (36%)	261 (35%)	78 (41%)	1.28 (0.93–1.78)	0.133	1.17 (0.77–1.78)	0.469		
Hypertension	362 (39%)	275 (37%)	87 (46%)	1.43 (1.04–1.98)	**0.028**	1.05 (0.70–1.59	0.805		
COPD	88 (9%)	64 (9%)	24 (13%)	1.53 (0.93–2.52)	0.094	0.63 (0.3–1.29)	0.204	0.61 (0.30–1.24)	0.179
Non-HCC cancer	113 (12%)	84 (11%)	29 (15%)	1.41 (0.89–2.23)	0.139	1.02 (0.48–2.16)	0.961		
HCC	69 (7%)	51 (7%)	18 (10%)	1.42 (0.89–2.23)	0.224	1.11 (0.57–2.15)	0.761		
Baseline laboratory values									
Creatinine (mg/dl)	0.87 (0.7-1.1)	0.86 (0.7-1.0)	0.92 (0.7-1.2)	1.19 (1.04–1.37)	**0.012**	1.10 (0.94–1.30)	0.237		
Bilirubin (mg/dl)	0.9 (0.5-1.7)	0.8 (0.5-1.3)	1.4 (0.8–3.3)	1.13 (1.08–1.18)	**<0.001**				
Serum albumin (g/dl)	3.7 (3.0-4.2)	3.8 (3.3-4.2)	3.1 (2.6-3.5)	0.90 (0.88–0.92)	**<0.001**				
Prothrombin time (s)	13 (11-15)	13 (11-15)	15 (13-17)	1.03 (1.01–1.05)	**0.005**				

Patient characteristics of CLD cohort with laboratory-confirmed SARS-CoV-2 infection including 70 patients with AIH. Univariable associations with death and associated *p* values assessed by logistic regression. Multivariable analysis for association with death was performed using logistic regression including all variables apart from bilirubin, albumin, and prothrombin time which form part of the Child-Turcotte-Pugh classification. Multivariable analysis demonstrated no associations between a diagnosis of AIH and death. Data was available for all patients in all categories (after applying the relevant assumptions for obesity and ethnicity) apart from missing data for creatinine in 70 (8%) bilirubin in 79 (8%), albumin 89 (10%), and prothrombin time 202 (22%). The absence or presence of AIH, NAFLD, ALD, HBV, or HCV was determined according to that reported by submitting clinician; a minority of patients had combinations of more than one liver disease aetiology except for variant syndromes of AIH which were excluded from the analysis. Patients who were reported by the submitting clinician to have a combination of liver disease aetiology, were classed as having more than one of NAFLD, ALD, HBV, or HCV in the analysis. AIH, autoimmune hepatitis; ALD, alcohol-related liver disease; CLD, chronic liver disease; COPD, chronic obstructive pulmonary disease; HCC, hepatocellular carcinoma; NAFLD, non-alcoholic fatty liver disease; OR, odds ratio.

Of the patients with AIH, major contributory countries included the USA 14 (21%), the UK 10 (15%), Spain 10 (15%), Iran 7 (10%), and Italy 7 (10%). Major comorbidities in the AIH cohort included hypertension 19 (27%), diabetes mellitus 11 (16%), heart disease 9 (13%), and COPD 3 (4%). Fifty-eight (83%) patients with AIH were taking immunosuppression; the immunosuppressive agents used were prednis(ol)one 41 (71%), thiopurines (azathioprine or 6-mercaptopurine) 32 (55%), mycophenolate mofetil (MMF) 9 (16%), tacrolimus 5 (9%), and budesonide 4 (7%). Thirty (52%) patients with AIH were on combined immunosuppression with ≥2 agents. For 19 (27%) patients with AIH, information was available regarding the modification of immunosuppression throughout the COVID-19 disease course. There were no changes to medications in 12 patients (63%), azathioprine was discontinued in 5 (26%), corticosteroid dosage was reduced in 2 (11%), and corticosteroid dosage was increased in 3 (16%).

### Period prevalence rates of SARS-CoV-2 in patients with AIH

In order to report on the unadjusted period prevalence of SARS-CoV-2 infection in the AIH population, 19 centres across the ERN RARE-LIVER network in Europe reported on the number of patients with AIH treated per year in addition to the number of cases of AIH testing positive for SARS-CoV-2. Up until 17^th^ July 2020, SARS-CoV-2 was detected in 20/3,043 (0.66%) patients with AIH. A similar period prevalence was observed in patients with PBC (12/3,314; 0.36%) and PSC (9/1,982; 0.45%).

### Non-CLD cohort

Within the same period, data were collected using an identical case report form for 793 consecutive non-CLD patients, with 769 cases remaining after exclusions (OUHFT 614; UNCH 155) (Fig. S1). The non-CLD cohort differed significantly from the AIH cohort with regards to age, sex, ethnicity, smoking status, baseline serum creatinine and rates of comorbidities including diabetes mellitus, hypertension, obesity and renal function (Table S4). The number of patients in the non-CLD cohort with unknown BMI who were assumed to be non-obese for the analysis was 158/769 (20%). The non-CLD cohort presented here includes 614 patients from OUHFT included in a previously published analysis comparing COVID-19 outcomes in liver transplant recipients.^7^

### Presenting signs and symptoms

Data on presenting symptoms were available for 65 (93%) patients in the AIH cohort, 677 (88%) in the non-CLD cohort, and 840 (97%) in the non-AIH CLD cohort. There were no differences between CLD patients with and without AIH in the proportions presenting with respiratory symptoms (74% *vs.* 77%; *p =* 0.546), gastrointestinal symptoms (26% *vs.* 22%; *p =* 0.441) and those who were asymptomatic (15% *vs.* 16%; *p =* 1.0). Compared to the non-CLD cohort, patients with AIH had a higher rate of gastrointestinal symptoms at presentation (26% *vs.* 14%; *p =* 0.016), but comparable rates of respiratory symptoms (74% *vs.* 83%; *p =* 0.016), and those who were asymptomatic (15% *vs.* 15%; *p =* 1.0).

### Outcomes

#### Hospitalization, intensive care unit admission and death in AIH cohort

**Fig. 2:**
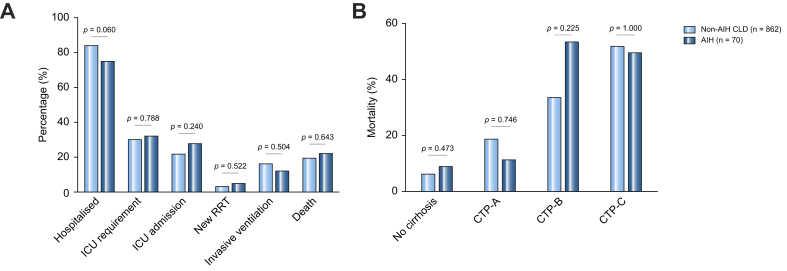
Rates of major outcomes in patients with AIH compared to other aetiologies of CLD and mortality between cohorts according to baseline liver disease severity. (A) Rates of major outcomes following SARS-COV-2 infection in patients with AIH compared to patients with non-AIH CLD. The discrepancy between the rates of ICU requirement and ICU admission are accounted for by a proportion of severe cases being deemed inappropriate for ICU admission or due to lack of ICU availability. (B) Comparison of mortality rates following SARS-COV-2 infection between AIH *vs.* non-AIH CLD separated by baseline liver disease severity: CLD without cirrhosis (9% *vs.* 7%; *p =* 0.473), Child-Pugh A (12% *vs.* 19%; *p =* 0.746), Child-Pugh B (54% *vs.* 34%; *p =* 0.225) Child-Pugh C (50% *vs.* 52%; *p =* 1.0). AIH, autoimmune hepatitis; CLD, chronic liver disease; ICU, intensive care unit; RRT, new requirement for renal replacement therapy; SARS-CoV-2, severe acute respiratory syndrome coronavirus 2.

When comparing AIH with non-AIH CLD there were no significant differences in the rates of all major outcomes including hospitalization (76% *vs.* 85%; *p =* 0.060), intensive care unit (ICU) requirement (33% *vs.* 31%; *p =* 0.788), ICU admission (29% *vs.* 23%; *p =* 0.240), new requirement for renal replacement therapy (6% *vs.* 4%; *p =* 0.522), invasive ventilation (13% *vs.* 17%; *p =* 0.504) and death (23% *vs.* 20%; *p =* 0.643) (Fig. 2A). The discrepancy between the rates of ICU requirement and ICU admission are accounted for by a proportion of severe cases being deemed inappropriate for ICU admission or due to the lack of ICU availability. Furthermore, rates of mortality did not differ between AIH and non-AIH CLD when stratified by baseline Child-Pugh class (Fig. 2B). Within the 16 patients with AIH who died, the major causes of death were COVID-19 lung disease 9 (56%), liver-related 5 (31%) and cardiac-related 2 (13%) and these did not differ significantly from those dying in the non-AIH CLD cohort. A summary of baseline characteristics and rates of major outcomes in patients with AIH separated by registry are reported in Table S5.

#### Characteristics and major outcomes in patients with variant syndromes

In the cohort of patients with variant syndromes and AIH with co-existing liver disease (n = 16) who were excluded from the analysis, 14 (88%) received immunosuppressive treatment, 10 (63%) had cirrhosis, 12 (75%) were hospitalized, and 4 (25%) died (Table S1). Within the cohort of 19 patients with PSC, 7 (37%) were on immunosuppressive treatment for inflammatory bowel disease, 9 (52%) had cirrhosis, 13 (57%) were hospitalized, and 4 (17%) died (Table S2). The PBC cohort included 19 patients of whom 17 (90%) were treated with ursodeoxycholic acid, 3 (16%) had cirrhosis, 6 (32%) were hospitalized, and 1 patient (5%) died (Table S3).

#### Factors associated with mortality within AIH cohort

Among patients with AIH, multivariable analysis of factors associated with death demonstrated positive associations with age (OR 2.01 per 10 years; 95% CI 1.07–3.81; *p =* 0.031), Child-Pugh B (OR 42.48; 95% CI 4.41–409.53; *p =* 0.001), and Child-Pugh C (OR 69.30; 95% CI 2.83–1694.50; *p =* 0.009) cirrhosis. However, there was no association between the use of immunosuppression and mortality. When backwards selection of variables was used with a threshold of *p* <0.2, the same factors remained significantly associated with death (Table 1). Data included in multivariable analysis was available for all patients in all categories.

#### Associations between AIH and mortality within total CLD cohort

Among the 932 cases in the total CLD cohort (including 70 patients with AIH), factors associated with death in multivariable analysis included age (OR 1.27; 95% CI 1.09–1.50; *p =* 0.003), ALD (OR 1.79; 95% CI 1.06–3.01; *p =* 0.018) and all stages of cirrhosis; Child-Pugh A (OR 2.18; 95% CI 1.24–3.84; *p =* 0.007), Child-Pugh B (OR 4.79; 95% CI 2.72–8.45; *p <*0.001), Child-Pugh C (OR 12.41; 95% CI 6.73–22.88; *p <*0.001). When backwards selection of variables was used with a threshold of *p <*0.2, the same factors remained significantly associated with death (Table 2). Specifically, a diagnosis of AIH was not associated with mortality. In addition, when a separate analysis was repeated to include only patients with AIH who were immunosuppressed as a variable, there remained no significant association with death (OR 1.27; 95% CI 0.49–3.34; *p =* 0.623). The total CLD cohort presented here includes 745 patients included in a previously published analysis, however this prior analysis did not consider AIH as a variable in any logistic regression models.^3^

#### Propensity score matched analysis comparing AIH with non-AIH CLD

**Fig. 3:**
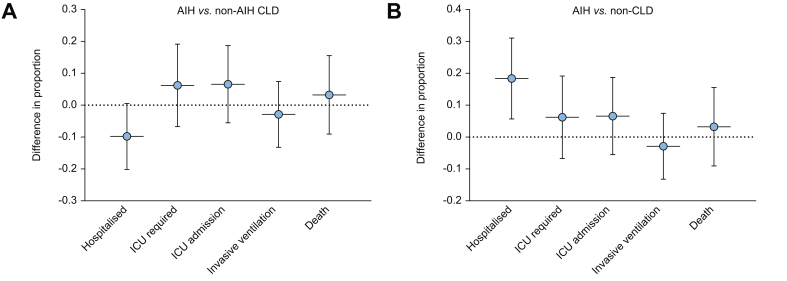
Propensity score matched analysis of major outcomes for AIH cohort compared with non-AIH CLD cohort and non-CLD cohort. (A) Plot shows propensity-score matched analyses for major outcomes following SARS-CoV-2 infection for AIH compared to non-AIH CLD. Variables selected for propensity score matching were age in years, interactions with age, sex, and baseline liver disease severity (CLD without cirrhosis, CTP-A, CTP-B, CTP-C). Bars represent 95% CIs. The risk of each major outcome between AIH *vs.* non-AIH CLD was hospitalization -9.7% (95% CI -20.3%–0.7%; *p =* 0.067), ICU requirement +6.2% (95% CI -0.07%–19.2%; *p =* 0.349), ICU admission +6.6% (95% CI -5.6%–18.8%; *p =* 0.289), invasive ventilation -2.9% (95% CI -13.3%–7.6%; *p =* 0.59, and death (+3.2%; 95% CI -9.2%–15.7%; *p =* 0.609). (B) Plot shows propensity-score matched analyses for major outcomes following SARS-CoV-2 infection for AIH compared to the non-CLD cohort. Variables selected for propensity score matching were age, interactions with age, sex, hypertension, COPD, heart disease and diabetes. The risk of each major outcome between AIH *vs.* non-CLD was hospitalization +18.4% (95% CI 5.6–31.2%; *p =* 0.005), ICU requirement +6.2% (95% CI -6.8%–19.3%; *p =* 0.349), ICU admission +6.6% (95% CI -5.6%–18.8%; *p =* 0.289), invasive ventilation -2.9% (95% CI -13.3%–7.5%; *p =* 0.590), and death +3.2% (95% CI -9.1%-15.6%; *p =* 0.609). AIH, autoimmune hepatitis; CLD, chronic liver disease; COPD, chronic obstructive pulmonary disease; ICU, intensive care unit; SARS-CoV-2, severe acute respiratory syndrome coronavirus 2.

To further investigate associations between AIH and major outcomes, a propensity score matched model was constructed including the variables age, interactions with age, sex, and baseline liver disease severity (CLD without cirrhosis, Child-Pugh A, Child-Pugh B, Child-Pugh C) to compare AIH with non-AIH CLD. Using this model, AIH conferred no additional risk compared with non-AIH CLD across all major outcomes including hospitalization, ICU admission, and death (Fig. 3A). Patient characteristics for the non-AIH CLD cohort after propensity score matching are presented in Fig. S6.

#### Propensity score matched analysis comparing AIH with non-CLD cohort

A propensity score matched analysis was also performed comparing rates of major outcomes for AIH *vs.* the non-CLD cohort derived from OUHFT (UK) and UNCH (USA) during the same period. Variables selected for propensity score matching included age, interactions with age, sex, hypertension, COPD, heart disease and diabetes. This demonstrated a significant increased risk of hospitalization for patients with AIH (+18.4% 95% CI 5.6–31.2%; *p =* 0.005), but no increased risk of all other major outcomes including ICU admission, and death (Fig. 3B). Patient characteristics for the non-CLD cohort after propensity score matching are presented in Fig. S6.

## Discussion

Identifying patient groups at high risk of SARS-CoV-2 infection, or those likely to have a severe clinical course of COVID-19 is essential in order to inform treatment decisions and infection prevention strategies. Whilst large population studies using electronic health records have significantly advanced our understanding of the risks posed by certain comorbidities,^2^ determining the impact of rare conditions on COVID-19 outcomes often requires large-scale clinician reporting of individual cases. This work is the first to characterise the disease course following SARS-CoV-2 infection in patients with AIH, a subpopulation where outcomes from COVID-19 remain poorly defined.

Within our combined registry dataset, multivariable analysis of 70 patients with AIH showed that only advancing age and baseline decompensated cirrhosis were independent risk factors for death. Importantly there was no association between mortality and the use of immunosuppression. Furthermore, we detected no association between AIH and death in a multivariable analysis of patients with CLD, and demonstrated comparable outcomes between AIH and propensity score matched cohorts without liver disease, and with CLD of other aetiology. The rates of major outcomes and lack of an association between immunosuppression and death in those with AIH is consistent with similar findings in the liver transplant population.^7^^,^^8^ The reasons for this are currently unknown, but presumably local or systemic immunity against SARS-CoV2 infection is preserved in spite of immunosuppressive medication. This contrasts with poor outcomes observed in patients with advanced liver disease where cirrhosis-associated immune dysfunction and clinical frailty may enhance disease susceptibility.

Up until this work, only small case series of patients with AIH and COVID-19 have been published. In a report from across several regions in Italy, 10 patients with AIH on immunosuppressive treatment showed a clinical course of COVID-19 comparable to that of non-immunosuppressed patients.^16^ Furthermore, telephone-based surveys in Northern Italy did not detect increased COVID-19 mortality in patients with AIH.^21^ These publications were important early signals for patients and their clinicians, but interpretations have been limited by very small sample sizes and localisation to one geographical area. This has led to difficulties in formulating clear recommendations on the approach to social distancing and use of immunosuppressive medication during the pandemic, which has by extension caused significant anxiety and uncertainty for patients. Although our data shows that patients with AIH are not more susceptible to death, many of these patients are currently completely avoiding social contact or “shielding”, which may reduce exposure to high SARS-CoV-2 viral load infections associated with worse clinical outcomes. It is now critical to vaccinate these patients against SARS-CoV-2 infection, and to monitor their response to immunisation which may be attenuated by immunosuppressive therapy.

Of note, we did observe greater rates of hospitalisation in patients with AIH compared to a matched cohort without liver disease. This may relate to patients with AIH being generally more likely to be hospitalized compared to patients without liver disease and therefore subject to higher rates of routine inpatient SARS-CoV-2 testing. In addition, it may reflect a background level of patient and clinician anxiety regarding the uncertain impact of immunosuppression on the COVID-19 disease course leading to a lower threshold for hospital admission. These anxieties can also be inferred from the fact that azathioprine maintenance therapy was discontinued in 25% of patients with AIH following SARS-CoV-2 infection. However, we do not have follow-up clinical data to assess the secondary impact of these medication changes on liver disease activity, including the rates of AIH flares.

The strengths of the current study include the international nature of case submissions which gives a truly global perspective on the impact of SARS-CoV-2 infection in patients with AIH. Clinician reporting also minimises the risk of misclassification although we accept that centralised diagnostic criteria for cirrhosis are lacking and that assessment of Child-Pugh class includes subjective components. An additional strength is the comparison of AIH cases with a matched group of contemporaneous patients without liver disease from the UK and USA (the 2 largest contributors of AIH cases), which strengthens the argument that these patients are not at higher risk of adverse COVID-19 outcomes. However, our findings must be interpreted in the context of the study’s potential limitations. Despite being the largest collection of patients with AIH and SARS-CoV-2 to date, the total numbers remain relatively small, constraining the number of variables included in propensity score matching and limiting interpretations with respect to variables associated with death within the AIH cohort. Secondly, registry data is vulnerable to reporting bias, leading to over-representation of patients with more severe liver disease and more severe COVID-19, and it is noteworthy that most case submissions were derived from tertiary care centres Tables S7 and S8). The high rates of cirrhosis in patients with AIH (54%) found in the current study may be an indication of this reporting bias given that previous studies have described a cirrhosis rate of 20–30% in those with AIH.^22^^,^^23^ However, despite the inclusion of patients with more severe baseline liver disease, we were still unable to identify an increased risk of severe COVID-19 and death in the AIH cohort, suggesting that the true risk is likely to be even lower than reported here. It is also notable that despite the study having international reach, the AIH cohort is predominantly derived from western populations which may limit external generalisability of the results to other geographical areas. Similarly, the classification of what constitutes an ‘intensive care unit’ and the parameters used to determine the clinical requirement for ICU are likely to vary between international institutions. Lastly, although a time-dependent analysis (*e.g*. Cox regression and Kaplan-Meier curves) may have been preferable over logistic regression in evaluating mortality, this approach was not possible due to the lack of accurate data on the duration from positive laboratory COVID-19 diagnosis to death in the COVID-Hep/SECURE cirrhosis registries.

In summary, this study involving more than 1,700 patients, helps characterize the COVID-19 disease course and risk of adverse outcome in 70 patients with AIH. Through multiple comparisons with non-AIH CLD and non-CLD cohorts, we demonstrate that AIH does not confer major additional susceptibility to adverse outcomes following SARS-COV-2 infection despite the potential reporting of cases with more severe liver disease. In this patient group, age and baseline liver disease severity remain the most important determinants of outcome in contrast to the use of immunosuppression for which no negative impact was detected. This should provide some reassurance to patients and clinicians and lends weight to recommendations that immunosuppressive medication should not routinely be modified or discontinued during the course of COVID-19.

### Abbreviations

AASLD, American Association for the Study of Liver Diseases; AIH, autoimmune hepatitis; ALD, alcohol-related liver disease; CLD, chronic liver disease; COPD, chronic obstructive pulmonary disease; COVID-19, coronavirus disease 2019; EASL, European Association for Study of the Liver; ICU, intensive care unit; OR, odds ratio; OUHFT, Oxford University Hospitals NHS Foundation Trust; PBC, primary biliary cholangitis; PSC, primary sclerosing cholangitis; PSM, propensity score matched; SARS-CoV-2, severe acute respiratory syndrome coronavirus 2; UNCH, University of North Carolina Hospitals.

## Financial support

The R-LIVER COVID-19 registry was supported by the European Reference Network on Hepatological Diseases (ERN RARE-LIVER). The COVID-Hep.net registry was supported by the 10.13039/501100009253European Association for the Study of the Liver (EASL) (2020RG03). SECURE-cirrhosis was also supported by the 10.13039/100000002National Institutes of Health grant T32 DK007634 (AMM), 10.13039/100011485North Carolina Translational and Clinical Sciences Institute (CTSA grant number UL1TR002489) and 10.13039/100000002National Institutes of Health (UL1TR002489). TM is supported by the 10.13039/100004440Wellcome Trust as a Clinical Research Training Fellow (102176/B/13/Z). EB is supported by the Oxford NIHR Biomedical Research Centre and is an NIHR Senior Investigator. The views expressed in this article are those of the authors and not necessarily those of EASL, ERN RARE-LIVER, the NHS, the NIHR, or the Departments of Health.

## Authors’ contributions

Concept and set-up of registries: TM, GB, MS, GJW, AL, AMM, EB, ASB, CC, CS. Concept and design of registry collaboration: TM, GB, MS, AL, GJW, AMM. Acquisition of AIH data: All authors. Acquisition of CLD data: TM, AMM, GJW, ASB, EB, MA. Acquisition of non-CLD cohort data: TM, CM, JK, LB, GJW, AMM. Statistical analysis: TM, GJW, GM, AKO. All authors were involved in critical review of the manuscript.

## Conflict of interest

The authors do not have any conflicts of interest.

Please refer to the accompanying ICMJE disclosure forms for further details.

## Data Availability

Data may be made available upon request to the corresponding authors.
