# Supplementary material for: SARS-CoV-2 infection in patients with autoimmune hepatitis
Source: J Hepatol. 2021 Jun;74(6):1335–43. doi: 10.1016/j.jhep.2021.01.021 (PMC7835076; doi:10.1016/j.jhep.2021.01.021)
Supplement: Multimedia component 1 [file mmc1.pdf]

Supplementary annex – COVID-Hep and SECURE-Cirrhosis case report form

Inclusion Criteria:

1) Chronic liver disease or post-liver transplantation AND

2) Laboratory confirmed COVID 19 infection

Ideally this form should be completed after the patient has had COVID 19 for a long enough duration to experience complete recovery, discharge, or death.

If you have any questions, please reach out to info@covid-hep.net

Reporter Information

Name of reporter	________________________
Email address of reporter	________________________
Name of lead physician providing care for liver disease/post-liver transplant	________________________
Name of center providing care for liver disease/post-liver transplant	________________________
Name of hospital where patient received care for COVID 19 (enter 'NA' if patient not hospitalized)	________________________

Patient Information

Is the patient >90 years of age?	◯ Yes<br>◯ No
Age	Drop down options
Country of residence	Drop down options

State of residence	◯ ALABAMA ◯ ALASKA<br>◯ AMERICAN SAMOA ◯ ARIZONA<br>◯ ARKANSAS ◯ CALIFORNIA<br>◯ COLORADO ◯ CONNECTICUT<br>◯ DELAWARE ◯ DISTRICT OF COLUMBIA<br>◯ FLORIDA ◯ GEORGIA<br>◯ GUAM ◯ HAWAII ◯ IDAHO<br>◯ ILLINOIS ◯ INDIANA<br>◯ IOWA ◯ KANSAS ◯ KENTUCKY<br>◯ LOUISIANA ◯ MAINE<br>◯ MARYLAND ◯ MASSACHUSETTS<br>◯ MICHIGAN ◯ MINNESOTA<br>◯ MISSISSIPPI ◯ MISSOURI<br>◯ MONTANA ◯ NEBRASKA<br>◯ NEVADA ◯ NEW HAMPSHIRE<br>◯ NEW JERSEY ◯ NEW MEXICO<br>◯ NEW YORK ◯ NORTH CAROLINA<br>◯ NORTH DAKOTA ◯ OHIO<br>◯ OKLAHOMA ◯ OREGON<br>◯ PENNSYLVANIA ◯ PUERTO RICO<br>◯ RHODE ISLAND ◯ SOUTH CAROLINA<br>◯ SOUTH DAKOTA ◯ TENNESSEE<br>◯ TEXAS ◯ UTAH ◯ VERMONT<br>◯ VIRGIN ISLANDS ◯ VIRGINIA<br>◯ WASHINGTON ◯ WEST VIRGINIA<br>◯ WISCONSIN ◯ WYOMING
Gender	◯ Female<br>◯ Male<br>◯ Other
Race/Ethnicity (may check more than one)	☐ White<br>☐ Black or African American<br>☐ American Indian / Native Alaskan<br>☐ East Asian (incl. Chinese, Japanese, Korean)<br>☐ South / South-East Asian (incl. Bangladeshi, Indian, Pakistani, Sri Lankan)<br>☐ Native Hawaiian / Pacific Islander<br>☐ Arabic<br>☐ Other<br>☐ Unknown
Other race/ethnicity	______________________________
Hispanic ethnicity	◯ Hispanic/Latino<br>◯ Not Hispanic/Latino<br>◯ Unknown
Patient BMI category (in kg/m2)	◯ < 18.5 (Underweight)<br>◯ 18.5-24.9 (Normal weight)<br>◯ 25.0-29.9 (Pre-obesity)<br>◯ 30.0-34.9 (Obesity class I)<br>◯ 35.0-39.9 (Obesity class II)<br>◯ >39.9 (Obesity class III)<br>◯ Unknown

Liver transplantation questions

Has the patient had a liver transplantation?

◯ Yes
◯ No

What year was the liver transplantation performed?	◯ Unknown<br>◯ 2020<br>◯ 2019<br>◯ 2018<br>◯ 2017<br>◯ 2016<br>◯ 2015<br>◯ 2014<br>◯ 2013<br>◯ 2012<br>◯ 2011<br>◯ 2010<br>◯ 2009<br>◯ 2008<br>◯ 2007<br>◯ 2006<br>◯ 2005<br>◯ 2004<br>◯ 2003<br>◯ 2002<br>◯ 2001<br>◯ 2000<br>◯ 1999<br>◯ 1998<br>◯ 1997<br>◯ 1996<br>◯ 1995<br>◯ 1994<br>◯ 1993<br>◯ 1992<br>◯ 1991<br>◯ 1990<br>◯ 1989<br>◯ 1988<br>◯ 1987<br>◯ 1986<br>◯ 1985<br>◯ 1984<br>◯ 1983<br>◯ 1982<br>◯ 1981<br>◯ 1980<br>◯ 1979<br>◯ 1978<br>◯ 1977<br>◯ 1976<br>◯ 1975<br>◯ 1974<br>◯ 1973<br>◯ 1972<br>◯ 1971<br>◯ 1970<br>◯ 1969<br>◯ 1968<br>◯ 1967<br>◯ 1966<br>◯ 1965
Indication for liver transplant (select all that apply)	☐ Decompensated cirrhosis<br>☐ Hepatocellular carcinoma<br>☐ Acute liver failure<br>☐ Other

Other indication for liver transplantation	______________________________
Underlying aetiology of liver disease (select all that apply)	☐ Non-alcoholic fatty liver disease (NAFLD)<br>☐ Alcohol-related liver disease (ALD)<br>☐ Hepatitis C virus (HCV)<br>☐ Hepatitis B virus (HBV)<br>☐ Autoimmune hepatitis (AIH)<br>☐ IgG4-related disease<br>☐ Primary biliary cholangitis (PBC)<br>☐ Primary sclerosing cholangitis (PSC)<br>☐ Hemochromatosis<br>☐ Wilson's disease<br>☐ Other
Other aetiology	______________________________
Immunosuppression regimen at time of COVID 19 infection (select all that apply)	☐ Prednisone<br>☐ Tacrolimus<br>☐ Sirolimus<br>☐ Everolimus<br>☐ Cyclosporine<br>☐ Mycophenolate mofetil (MMF)<br>☐ Azathioprine<br>☐ Cyclophosphamide<br>☐ Other<br>☐ Unknown
What other immunosuppression medication was used at the time of COVID 19 infection?	______________________________
Current prophylactic antimicrobial regimen (select all that apply)	☐ Trimethoprim/sulfamethoxazole or Co-trimoxazole<br>☐ Dapsone<br>☐ Pentamidine<br>☐ Acyclovir/valacyclovir<br>☐ Fluconazole<br>☐ Ganciclovir/valganciclovir<br>☐ Foscarnet<br>☐ Other<br>☐ Unknown<br>☐ None
Other prophylactic antimicrobial regimen	______________________________

Does the patient have any of the following comorbidities (check all that apply)?	☐ Cardiovascular disease (coronary artery disease, heart failure, arrhythmia, etc.)<br>☐ Diabetes<br>☐ Asthma<br>☐ COPD<br>☐ Other Chronic Lung Disease (NOT asthma/COPD)<br>☐ Hypertension<br>☐ Non-HCC cancer<br>☐ History of stroke<br>☐ Chronic renal disease (CKD, etc.)<br>☐ Human immunodeficiency virus (HIV) infection<br>☐ Current cigarette smoker<br>☐ Current user of tobacco products other than cigarettes (vaping, etc)<br>☐ Current heavy alcohol use (>2 drinks/day for men, >1 drink/day for women)<br>☐ History of illicit drug use including injectable drugs or inhaled crack/cocaine but excluding marijuana

Chronic liver disease questions

Aetiology of liver disease (select all that apply)	☐ Non-alcoholic fatty liver disease (NAFLD)<br>☐ Alcohol-related liver disease (ALD)<br>☐ Hepatitis C virus (HCV)<br>☐ Hepatitis B virus (HBV)<br>☐ Autoimmune hepatitis (AIH)<br>☐ IgG4-related disease<br>☐ Primary biliary cholangitis (PBC)<br>☐ Primary sclerosing cholangitis (PSC)<br>☐ Hemochromatosis<br>☐ Wilson's disease<br>☐ Other
Other aetiology	__________________________________
Does the patient have cirrhosis?	◯ Yes<br>◯ No<br>◯ Unknown
Child Pugh grade prior to COVID 19 diagnosis?	◯ A<br>◯ B<br>◯ C<br>◯ Unknown
Did the patient have ascites prior to COVID 19 diagnosis?	◯ None<br>◯ Mild/moderate (diuretic responsive)<br>◯ Severe (diuretic refractory)<br>◯ Unknown

What was the worst grade of hepatic encephalopathy patient developed prior to COVID 19 diagnosis?	◯ None<br>◯ Grade 1 (trivial lack of awareness, shortened attention span)<br>◯ Grade 2 (lethargy, minimal disorientation, subtle personality change)<br>◯ Grade 3 (somnolence to semi-stupor but responsive to verbal stimuli, gross disorientation)<br>◯ Grade 4 (coma - unresponsive to verbal or noxious stimuli)<br>◯ Unknown
Has the patient ever had hepatocellular carcinoma?	◯ Yes<br>◯ No<br>◯ Unknown
What immunosuppression was the patient taking for IgG4-related disease at time of COVID 19 diagnosis (may check more than one)?	☐ None<br>☐ Corticosteroids<br>☐ Azathioprine<br>☐ Rituximab<br>☐ Other<br>☐ Unknown
Other immunosuppression for IgG4-related disease	______________________________
Has the patient received steroids for alcoholic hepatitis recently (within 4 weeks of COVID 19 diagnosis)?	◯ Yes<br>◯ No<br>◯ Unknown
Treatment for primary biliary cholangitis (PBC) at time of COVID 19 diagnosis	☐ Ursodeoxycholic acid<br>☐ Obeticholic acid<br>☐ Fibrate<br>☐ Other<br>☐ No treatment<br>☐ Unknown
Other PBC treatments	______________________________
Does the patient have inflammatory bowel disease (IBD)?	◯ Yes<br>◯ No<br>◯ Unknown
Immunosuppression medication(s) patient was taking for IBD at time of COVID 19 diagnosis	☐ Prednisone/prednisolone<br>☐ Budesonide<br>☐ Azathioprine<br>☐ Methotrexate<br>☐ Mycophenolate<br>☐ Infliximab<br>☐ Adalimumab<br>☐ Ustekinumab<br>☐ Vedolizumab<br>☐ Other<br>☐ None<br>☐ Unknown
Other immunosuppression for IBD	______________________________

Treatment for autoimmune hepatitis (AIH) at time of COVID 19 diagnosis	☐ Prednisone/prednisolone<br>☐ Budesonide<br>☐ Azathioprine<br>☐ Mycophenolate<br>☐ Tacrolimus<br>☐ Other<br>☐ None<br>☐ Unknown
Other treatment for AIH	________________________________
Hepatitis B surface antigen (HBsAg) positive	○ Yes<br>○ No<br>○ Unknown
Treatment for HBV at the time of COVID 19 diagnosis	○ Tenofovir<br>○ Entecavir<br>○ Interferon<br>○ None<br>○ Unknown
Did the patient have detectable hepatitis C virus (HCV) RNA at the time of or prior to COVID 19 diagnosis?	○ Yes<br>○ No<br>○ Unknown
HCV genotype	○ 1<br>○ 2<br>○ 3<br>○ 4<br>○ 5<br>○ 6<br>○ 7<br>○ Unknown
Active treatment for HCV at the time of COVID 19 diagnosis	○ Yes<br>○ No<br>○ Unknown
Does the patient have any of the following comorbidities (check all that apply)?	☐ Cardiovascular disease (coronary artery disease, heart failure, arrhythmia, etc.)<br>☐ Diabetes<br>☐ Asthma<br>☐ COPD<br>☐ Other Chronic Lung Disease (NOT asthma/COPD)<br>☐ Hypertension<br>☐ Non-HCC cancer<br>☐ History of stroke<br>☐ Chronic renal disease (CKD, etc.)<br>☐ Human immunodeficiency virus (HIV) infection<br>☐ Current cigarette smoker<br>☐ Current user of tobacco products other than cigarettes (vaping, etc)<br>☐ Current heavy alcohol use (>2 drinks/day for men, >1 drink/day for women)<br>☐ History of illicit drug use including injectable drugs or inhaled crack/cocaine but excluding marijuana

Laboratory data (leave fields blank if unknown)

- **If COVID-19 suspected at presentation/admission then use recent pre-admission laboratory values as baseline.**
- **If hospital acquired COVID-19 suspected then please use first laboratory values obtained during hospitalisation as baseline.**

Baseline serum sodium (mmol/L)	________________ (Before COVID 19 infection)
Nadir serum sodium (mmol/L)	________________ (During COVID 19 infection)
Creatinine units	○ µmol/L ○ mg/dl
Baseline serum creatinine	________________ (Before COVID 19 infection)
Peak serum creatinine	________________ (During COVID 19 infection)
Baseline prothrombin time (sec)	________________ (Before COVID 19 infection)
Peak prothrombin time (sec)	________________ (During COVID 19 infection)
Albumin units	○ g/dl ○ g/liter
Baseline albumin	________________ (Before COVID 19 infection)
Nadir albumin	________________ (During COVID 19 infection)
Total bilirubin units	○ µmol/L ○ mg/dl
Baseline total bilirubin	________________ (Before COVID 19 infection)
Peak total bilirubin	________________ (During COVID 19 infection)
Baseline alanine aminotransferase (ALT) (IU/L)	________________ (Before COVID 19 infection)

Peak alanine aminotransferase (ALT) (IU/L)	____________ (During COVID 19 infection)
Baseline alkaline phosphatase (IU/L)	____________ (Before COVID 19 infection)
Peak alkaline phosphatase (IU/L)	____________ (During COVID 19 infection)

COVID 19 questions

Was this lab confirmed COVID 19 infection?	◯ Yes<br>◯ No<br>◯ Unknown
What symptoms did the patient have at the time of COVID 19 diagnosis?	◯ GI symptoms (abdominal pain, diarrhea, nausea, vomiting)<br>◯ Respiratory symptoms (shortness of breath, cough)<br>◯ Both GI and respiratory symptoms<br>◯ Neither GI or respiratory symptoms<br>◯ Unknown
Did patient test positive for influenza at time of COVID 19 infection?	◯ Yes<br>◯ No<br>◯ Unknown
What complications did the patient develop during COVID 19 infection?	☐ New or worsening ascites<br>☐ Spontaneous bacterial peritonitis<br>☐ Hepatic encephalopathy<br>☐ Non-variceal upper GI bleeding<br>☐ Variceal upper GI bleeding<br>☐ New requirement for renal replacement therapy (e.g. hemodialysis)<br>☐ Other
Other complications of COVID 19 infection	____________
What was the grade of ascites during COVID 19 infection?	◯ Mild/moderate<br>◯ Severe<br>◯ Unknown
What was the worst grade of hepatic encephalopathy during COVID 19 infection (based on West Haven Criteria)?	◯ Grade 1 (trivial lack of awareness, shortened attention span)<br>◯ Grade 2 (lethargy, minimal disorientation, subtle personality change)<br>◯ Grade 3 (somnolence to semi-stupor but responsive to verbal stimuli, gross disorientation)<br>◯ Grade 4 (coma - unresponsive to verbal or noxious stimuli)<br>◯ Unknown
Did the patient receive specific antiviral treatment for COVID 19 infection?	◯ Yes<br>◯ No<br>◯ Unknown

Which of the following treatment(s) did patient receive for COVID 19 (select all that apply)?	☐ Remdesivir<br>☐ Tocilizumab<br>☐ Lopinovir/ritonavir<br>☐ Chloroquine/hydroxychloroquine<br>☐ Ribavirin<br>☐ Interferon-alpha<br>☐ Other<br>☐ Unknown
Other type of antiviral treatment(s) received for COVID-19	________________________
What was the reason the patient did not receive COVID 19 antiviral treatment (select all that apply)?	☐ Elevated liver enzymes<br>☐ Underlying liver fibrosis/cirrhosis<br>☐ Other contraindication (eg AKI)<br>☐ Treatment not available<br>☐ Other<br>☐ Unknown
What was the reason the patient did not receive COVID 19 antiviral treatment?	________________________
Did the patient die?	◯ Yes<br>◯ No<br>◯ Unknown
What was the primary cause of death?	◯ Liver-related complications<br>◯ COVID 19-related lung disease<br>◯ Cardiogenic shock<br>◯ Other
Other cause of death	________________________
Has the patient been hospitalized?	◯ Yes<br>◯ No<br>◯ Unknown
Has the patient been discharged from the hospital?	◯ Yes<br>◯ No<br>◯ Unknown
Length of hospital stay (days)	________________________
Did patient receive invasive ventilation?	◯ Yes<br>◯ No<br>◯ Unknown
Did the patient receive non-invasive ventilation?	◯ Yes<br>◯ No<br>◯ Unknown
Was the patient admitted to an intensive care unit?	◯ Yes<br>◯ No<br>◯ Unknown

Why was the patient not admitted to an intensive care unit?	◯ Disease not severe enough<br>◯ Disease was severe enough but limited availability of intensive care unit<br>◯ Disease was severe enough but escalation to intensive care unit not felt to be appropriate.<br>◯ Unknown
