# Supplementary material for: SARS-CoV-2 infection in patients with autoimmune hepatitis
Source: J Hepatol. 2021 Jun;74(6):1335–43. doi: 10.1016/j.jhep.2021.01.021 (PMC7835076; doi:10.1016/j.jhep.2021.01.021)
Supplement: Multimedia component 2 [file mmc2.pdf]

R-LIVER COVID-19 - version 35.41

Printed on 08-07-2020 18:06:08 by Gustav Buescher

1. Baseline - Contributer

Number	Question	Answers
1.1	Name of contributer	
1.2	Email of contributer	
1.3	Has the case been integrated into the COVID-HEP registry?	◯ yes<br>◯ no

2. Baseline - Patient

Number	Question	Answers
2.1	Country	
2.2	Age	years
2.3	Sex	◯ male<br>◯ female<br>◯ other
2.4	Weight	kg
2.2.1	***If 'Age' is smaller or equal than '18' answer this question:***<br>Z-score weight	
2.5	Height	cm
2.2.2	***If 'Age' is smaller or equal than '18' answer this question:***<br>Z-score height	

2.6	BMI	
2.7	Smoking	◯ yes<br>◯ no
2.7.1	***If 'Smoking' is equal to 'yes' answer this question:***<br>Amount	[ ] pack years
2.8	Comorbidities	☐ COPD<br>☐ Asthma<br>☐ Diabetes<br>☐ Hypertension<br>☐ Cardiovascular disease<br>☐ other pulmonary disease<br>☐ other
2.8.1	***If 'Comorbidities' is equal to 'Cardiovascular disease' answer this question:***<br>Cardiovascular disease	[ ]
2.8.2	***If 'Comorbidities' is equal to 'other pulmonary disease' answer this question:***<br>Pulmonary disease	[ ]
2.8.3	***If 'Comorbidities' is equal to 'other' answer this question:***<br>Other disease	[ ]
2.9	Medication	[ ]

3. Baseline - Liver disease

Number	Question	Answers
3.1	Diagnosis	◯ Autoimmune Hepatitis<br>◯ Liver transplantation<br>◯ PBC<br>◯ PSC

		◯ Biliary atresia<br>◯ IgG4-associated disease<br>◯ other
3.2	Date of underlying rare liver disease diagnosis	[ ] [ ] [ ] *(dd-mm-yyyy)*
3.1.1	***If 'Diagnosis' is equal to 'Liver transplantation' answer this question:***<br>Date of transplantation	[ ] [ ] [ ] *(dd-mm-yyyy)*
3.1.2	***If 'Diagnosis' is equal to 'Liver transplantation' answer this question:***<br>Reason for transplantation	[ ]
3.1.3	***If 'Diagnosis' is equal to 'PBC' answer this question:***<br>Date of PBC diagnosis	[ ] [ ] [ ] *(dd-mm-yyyy)*
3.1.4	***If 'Diagnosis' is equal to 'PSC' answer this question:***<br>Date of PSC diagnosis	[ ] [ ] [ ] *(dd-mm-yyyy)*
3.1.5	***If 'Diagnosis' is equal to 'PSC' answer this question:***<br>Inflammatory bowel disease ?	☐ yes<br>☐ no
3.1.5.1	***If 'Inflammatory bowel disease ?' is equal to 'yes' answer this question:***<br>specify IBD treatment	[ ]
3.1.6	***If 'Diagnosis' is equal to 'other' answer this question:***<br>specify Diagnosis	[ ]
3.3	Liver cirrhosis	◯ yes<br>◯ no
3.3.1	***If 'Liver cirrhosis' is equal to 'yes' answer this question:***<br>Child Pugh	☐ Child Pugh A<br>☐ Child Pugh B<br>☐ Child Pugh C
3.4	Known Hepatocellular Carcinoma (HCC)?	☐ yes<br>☐ no
3.5	Known Cholangiocellular Carcinoma?	☐ yes<br>☐ no
3.6	Immunosuppressive therapy	☐ Prednisolone<br>☐ Azathioprin<br>☐ Tacrolimus<br>☐ MMF/CellCept

		☐ Budesonide<br>☐ other
3.6.1	***If 'Immunosuppressive therapy' is equal to 'Prednisolone' answer this question:***<br>Prednisolone dose	[ ] mg
3.6.2	***If 'Immunosuppressive therapy' is equal to 'Azathioprin' answer this question:***<br>Azathioprin dose	[ ] mg
3.6.3	***If 'Immunosuppressive therapy' is equal to 'Tacrolimus' answer this question:***<br>Tacrolimus dose	[ ] mg
3.6.4	***If 'Immunosuppressive therapy' is equal to 'Tacrolimus' answer this question:***<br>Last Tacrolimus level	[ ]
3.6.5	***If 'Immunosuppressive therapy' is equal to 'Tacrolimus' answer this question:***<br>Date of last Tacrolimus level	[ ] [ ] [ ] *(dd-mm-yyyy)*
3.6.6	***If 'Immunosuppressive therapy' is equal to 'MMF/CellCept' answer this question:***<br>MMF/CellCept dose	[ ] mg
3.6.7	***If 'Immunosuppressive therapy' is equal to 'Budesonide' answer this question:***<br>Budesonide dose	[ ] mg
3.6.8	***If 'Immunosuppressive therapy' is equal to 'other' answer this question:***<br>Other medication dosis	[ ]

4. Baseline - Covid-19 infection

Number	Question	Answers
4.1	COVID-19 diagnosis	[ ] [ ] [ ] *(dd-mm-yyyy)*

4.2	How many days until negative COVID-19 PCR?	days
4.3	Adaptation of immunosuppressive therapy performed due to Covid-19 infection?	◯ yes<br>◯ no
4.3.1	***If 'Adaptation of immunosuppressive therapy performed due to Covid-19 infection?' is equal to 'yes' answer this question:***<br>Please specify adaptation	
4.4	Symptoms	☐ Fever<br>☐ Coughing<br>☐ Shortness of breath<br>☐ Fatigue<br>☐ Myalgia
4.4.1	***If 'Symptoms' is equal to 'Fever' answer this question:***<br>Start date fever	*(dd-mm-yyyy)*
4.4.2	***If 'Symptoms' is equal to 'Fever' answer this question:***<br>Days of fever	days
4.4.3	***If 'Symptoms' is equal to 'Coughing' answer this question:***<br>Start date coughing	*(dd-mm-yyyy)*
4.4.4	***If 'Symptoms' is equal to 'Coughing' answer this question:***<br>Days of coughing	days
4.4.5	***If 'Symptoms' is equal to 'Shortness of breath' answer this question:***<br>Start date shortness of breath	*(dd-mm-yyyy)*
4.4.6	***If 'Symptoms' is equal to 'Shortness of breath' answer this question:***<br>Days of shortness of breath	days
4.4.7	***If 'Symptoms' is equal to 'Fatigue' answer this question:***<br>Start date fatigue	*(dd-mm-yyyy)*
4.4.8	***If 'Symptoms' is equal to 'Fatigue' answer this question:***<br>Days of fatigue	days
	Clinical findings at diagnosis	
4.5	Oxygen saturation	

		%
4.6	Respiratory rate	breaths per minute
4.7	Heart rate	bpm
4.8	Blood pressure	mmHg
4.9	X-Ray/CT	
	Lab results at diagnosis	
4.10	WBC count	Mrd/l
4.11	CRP	mg/l
4.12	LDH	U/L
4.13	pCT	µg/l
4.14	AST	U/l
4.15	ALT	U/l
4.16	gGT	U/l
4.17	Alk. Phos.	U/l
4.18	Total Bilirubin	mg/dl
4.19	Survived infection	yes<br>no
4.19.1	***If 'Survived infection' is equal to 'yes' answer this question:***<br>End of infection	*(dd-mm-yyyy)*
4.19.2	***If 'Survived infection' is equal to 'no' answer this question:***<br>Date of death	*(dd-mm-yyyy)*
4.19.3	***If 'Survived infection' is equal to 'no' answer this question:***	

	Cause of death	

5. Baseline - Clinical course

Number	Question	Answers
	course of disease	
5.1	Follow-up performed?	◯ yes<br>◯ no
	Please add a record for each follow-up	
5.1.2	***If 'Follow-up performed?' is equal to 'yes' answer this question:***<br>Clinical data	

6. Baseline - Inpatient treatment

Number	Question	Answers
6.1	Inpatient care necessary	◯ yes<br>◯ no
6.1.1	***If 'Inpatient care necessary' is equal to 'yes' answer this question:***<br>Duration	days
6.1.2	***If 'Inpatient care necessary' is equal to 'yes' answer this question:***<br>Oxygen therapy necessary?	◯ yes<br>◯ no

6.1.2.1	***If 'Oxygen therapy necessary?' is equal to 'yes' answer this question:***<br>How many days was oxygen therapy necessary?	
6.1.3	***If 'Inpatient care necessary' is equal to 'yes' answer this question:***<br>Did the patient develop a bacterial superinfection (such as bacterial pneumonia)?	yes<br>no
6.1.3.1	***If 'Did the patient develop a bacterial superinfection (such as bacterial pneumonia)?' is equal to 'yes' answer this question:***<br>Please specify diagnosis and treatment	
6.1.4	***If 'Inpatient care necessary' is equal to 'yes' answer this question:***<br>Other complications at any time during hospitalisation?	
6.1.5	***If 'Inpatient care necessary' is equal to 'yes' answer this question:***<br>Dialysis	yes<br>no
6.1.6	***If 'Inpatient care necessary' is equal to 'yes' answer this question:***<br>Experimental therapy	Lopinavir/Ritonavir<br>Remdesivir<br>Hydroxychloroquin<br>Tocilizumab<br>other<br>none
6.1.6.1	***If 'Experimental therapy' is equal to 'other' answer this question:***<br>Please specify experimental therapy	

7. Baseline - Intensive care

Number	Question	Answers

7.1	Intensive care needed?	◯ yes<br>◯ no
7.1.1	***If 'Intensive care needed?' is equal to 'yes' answer this question:***<br>Duration	[ ] days
7.1.2	***If 'Intensive care needed?' is equal to 'yes' answer this question:***<br>ARDS	◯ yes<br>◯ no
7.1.2.1	***If 'ARDS' is equal to 'yes' answer this question:***<br>specify ARDS treatment and course	
7.1.3	***If 'Intensive care needed?' is equal to 'yes' answer this question:***<br>Ventilation necessary?	☐ NIV<br>☐ conventional invasive ventilation<br>☐ vvECMO<br>☐ vaECMO<br>☐ no
7.1.3.1	***If 'Ventilation necessary?' is equal to 'NIV' answer this question:***<br>Days of NIV	[ ] days
7.1.3.2	***If 'Ventilation necessary?' is equal to 'conventional invasive ventilation' answer this question:***<br>Days of conventional invasive ventilation	[ ] days
7.1.3.3	***If 'Ventilation necessary?' is equal to 'vvECMO' answer this question:***<br>Days of vvECMO	[ ] days
7.1.3.4	***If 'Ventilation necessary?' is equal to 'vaECMO' answer this question:***<br>Days of vaECMO	[ ] days
7.1.4	***If 'Intensive care needed?' is equal to 'yes' answer this question:***<br>Intensive care complications?	
