# Supplementary material for: SARS-CoV-2 infection in patients with autoimmune hepatitis
Source: J Hepatol. 2021 Jun;74(6):1335–43. doi: 10.1016/j.jhep.2021.01.021 (PMC7835076; doi:10.1016/j.jhep.2021.01.021)
Supplement: Multimedia component 3 [file mmc3.pdf]

SARS-CoV-2 infection in patients with autoimmune hepatitis

Thomas Marjot, Gustav Buescher, Marcial Sebode, Eleanor Barnes, Alfred S Barritt IV, Matthew J Armstrong, Luke Baldelli, James Kennedy, Carolyn Mercer, Ann-Kathrin Ozga, Christian Casar, Christoph Schramm, contributing Members and Collaborators of ERN RARE-LIVER / COVID-Hep / SECURE-Cirrhosis, Andrew M Moon, Gwilym J Webb, Ansgar W Lohse

Table of contents

Table S1..2

Table S2..3

Table S3..4

Table S4..5

Table S5..6

Table S6..7

Table S8..9

Fig. S1..20

Table S1. Patient characteristics and rates of major outcomes of patients with variant syndromes and AIIH with co-existing liver disease.

**Variant syndromes and AIH with co-existing liver disease**	**Number (n=16)**
AIH / PBC	7
AIH / NAFLD	3
AIH / HCV	1
AIH / ALD	1
AIH / PSC	3
AIH / PSC / IgG4	1
Median age (IQR)	45 (36–57)
Female	11 (69%)
Obesity	4 (25%)
Cirrhosis	10 (63%)
CTP A	5 (50%)
CTP B	4 (25%)
CTP C	1 (10%)
Immunosuppressive treatment	14 (88%)
Hospitalization	12 (75%)
ICU admission	4 (25%)
Death (%)	3 (30%)

Table S1: Patient characteristics of 16 patients with laboratory-confirmed SARS-CoV-2 infection and variant syndromes or AIH with co-existing liver disease. AIH = autoimmune hepatitis; ALD = alcohol related liver disease; HCV = chronic hepatitis C virus infection; ICU = intensive care unit; IgG4 = immunoglobulin G4-related disease; IQR = interquartile range; NAFLD = non-alcoholic fatty liver disease; Obesity = BMI >30kg/m^2; PBC, primary biliary cholangitis; PSC = primary sclerosing cholangitis.

Table S2. Patient characteristics and rates of major outcomes in PSC patients

**Characteristic / outcome**	**PSC (n=19)**
Median age (IQR)	56 (40-63)
Female	9 (47%)
Obesity	3 (16%)
IBD	10 (53%)
Immunosuppressive treatment for IBD	7 (37%)
Cirrhosis	9 (47%)
CTP A	5 (56%)
CTP B	2 (22%)
CTP C	2 (22%)
Hospitalization	11 (58%)
ICU admission	3 (16%)
Death	4 (21%)

Table S2: Patient characteristics of 19 PSC patients with laboratory-confirmed SARS-CoV-2 infection; CTP = Child-Turcotte-Pugh; IBD = inflammatory bowel disease; ICU = intensive care unit; IQR = interquartile range; Obesity = BMI >30kg/m^2; PSC = primary sclerosing cholangitis.

Table S3. Patient characteristics and rates of major outcomes in PBC patients

**Characteristic / outcome**	**PBC (n=19)**
Median age (IQR)	62 (53–76)
Female	16 (84%)
Obesity	1 (5%)
UDCA treatment	17 (90%)
Cirrhosis	3 (16%)
CTP A	1 (33%)
CTP B	1 (33%)
CTP C	1 (33%)
Hospitalization	6 (32%)
ICU admission	0
Death	1 (5%)

Table S3: Patient characteristics of 19 PBC patients with laboratory-confirmed SARS-CoV-2 infection; ICU = intensive care unit; IQR = interquartile range; Obesity = BMI >30kg/m^2; PBC = primary biliary cholangitis; UDCA = ursodeoxycholic acid.

Table S4. Characteristics of AIH cohort compared with non-CLD cohort

	**AIH (n=70)**	**non-CLD (n=769)**	**p-value**
Demographics			
Age	55 (44–71)	66 (50–82)	**<0.001**
Sex (female)	49 (70%)	373 (48.5%)	**<0.001**
Ethnicity (white)	33 (47%)	489 (64%)	**0.01**
Cofactors			
Diabetes mellitus	11 (16%)	46 (6%)	**0.005**
Hypertension	19 (27%)	75 (10%)	**<0.001**
COPD	3 (4%)	18 (2%)	0.409
Cancer	4 (6%)	22 (3%)	0.265
Smoker	2 (3%)	2 (<1%)	0.037
Obesity	11 (16%)	229 (30%)	**0.013**
Heart disease	9 (13%)	72 (9%)	0.395
non-HCC cancer	3 (4%)	22 (3%)	0.457
HCC	1 (1%)	0 (0%)	-
Creatinine (mg/dL)	0.87 (0.70–1.06)	0.89 (0.72–1.11)	**0.006**
Major outcomes			
Hospitalization	53 (76%)	508 (66%)	0.112
ICU requirement	23 (33%)	203 (26%)	0.261
ICU admission	20 (29%)	74 (10%)	**<0.001**
RRT	4 (6%)	5 (1%)	**0.004**
Invasive ventilation	9 (13%)	49 (6%)	**0.049**
Death	16 (23%)	166 (22%)	0.764

Table S4. Demographics, cofactors, and major outcomes of 70 AIH patients with SARS-COV-2 compared with contemporaneous cohort of 769 non-CLD patients testing positive for SARS-CoV-2 at Oxford University Hospitals NHS Foundation Trust (UK) and University of North Carolina Hospitals (USA). COPD = chronic obstructive pulmonary disease; HCC = hepatocellular carcinoma; ICU = intensive care unit; Obesity = BMI >30kg/m^2; RRT = new requirement for renal replacement therapy.

Table S5. Composition and characteristics of COVID-Hep/SECURE-Cirrhosis and R-Liver

	**COVID-Hep/SECURE-Cirrhosis**	**R-LIVER**
**AIH (n)**	**51**	**14**
Female	37 (73%)	9 (64%)
Median age years (IQR)	54 (43–66)	62 (44–72)
Cirrhosis	30 (59%)	6 (43%)
Hospitalization	40 (78%)	9 (64%)
ICU admission	16 (31%)	3 (21%)
Death	14 (28%)	1 (7%)
**PBC (n)**	**8**	**11**
Female	7 (87.5%)	9 (82%)
Median age years (IQR)	60.5 (49–81)	63 (56–70)
Cirrhosis	2 (25%)	1 (9%)
Hospitalization	4 (50%)	2 (18%)
ICU admission	0	0
Death	0	1 (9%)
**PSC (n)**	**14**	**3**
Female	7 (50%)	1 (33%)
Median age years (IQR)	54 (39–69)	57 (56–63)
Cirrhosis	7 (50%)	2 66%)
Hospitalization	8 (57%)	2 (66%)
ICU admission	3 (21%)	0
Death	4 (29%)	0

Table S5: Composition of COVID-Hep/SECURE-Cirrhosis and R-Liver registries. 7 Patients (5 AIH and 2 PSC patients) were reported to both registries which were included individually in the main analysis but are excluded from this table. AIH = autoimmune hepatitis; ICU = intensive care unit; IQR = interquartile range; PBC, primary biliary cholangitis; PSC, Primary sclerosing cholangitis

Table S6. Patient characteristics after propensity score matching

Characteristic	AIH (n=70)	Non-AIH CLD (n=140)	non CLD (n=140)
Demographics			
Age (years)	55 (44–71)	55 (41–71)	52 (42–62)
Sex (male)	21 (30%)	42 (30%)	42 (30%)
Ethnicity (white)	33 (47%)	64 (46%)	78 (56%)
Liver disease severity			
CLD without cirrhosis	32 (46%)	71 (51%)	-
CTP-A	17 (24%)	31 (22%)	-
CTP-B	13 (19%)	31 (22%)	-
CTP-C	8 (11%)	7 (5%)	-
Aetiology			
AIH	70 (100%)	0 (0%)	-
NAFLD	0 (0%)	64 (46%)	-
ALD	0 (0%)	22 (16%)	-
HCV	0 (0%)	25 (18%)	-
HBV	0 (0%)	19 (14%)	-
Co-factors			
Smoker	2 (3%)	8 (6%)	0 (0%)
Obesity	11 (16%)	35 (25%)	48 (34%)
Heart disease	9 (13%)	18 (13%)	8 (6%)
Diabetes mellitus	11 (16%)	57 (41%)	15 (11%)
Hypertension	19 (27%)	54 (39%)	29 (21%)
COPD	3 (4%)	9 (7%)	0 (0%)
non-HCC cancer	3 (4%)	10 (7%)	5 (4%)
HCC	8 (6%)	1 (1%)	0 (0%)
Creatinine (mg/dl)	0.8 (0.6–1.0)	0.8 (0.6–1.1)	0.8 (0.7–1.0)

Table S6. Table shows the baseline patient characteristics for non-AIH CLD cohort and non-CLD cohort after performing propensity score matching to non-CLD patients. Variables selected for propensity score matching in the non-AIH CLD cohort were age, interactions with age, sex, and baseline liver disease severity (CLD without cirrhosis, CTP-A, CTP-B, CTP-C). Variable selected for propensity score matching in the non-CLD cohort were age, interactions with age, sex, hypertension, COPD, heart disease and diabetes. HCC = hepatocellular carcinoma; COPD = chronic obstructive pulmonary disease; CTP = Child-Turcotte-Pugh; NAFLD = non-alcoholic fatty liver disease; ALD = Alcohol related liver disease; HBV = chronic hepatitis B virus infection; HCV = chronic hepatitis C virus infection;

Table S7. List of members and collaborators of ERN RARE-LIVER

Collaborator	Centre
Annika Bergquist	Karolinska University Hospital; Stockholm, Sweden
Alessio Gerussi	Azienda Sanitaria Socio Assistenziale (ASST)-Monza, Ospedale San Gerardo; Monza, Italy
Maria-Carlota Londoño, Albert Pares	Hospital Clínic i Provincial de Barcelona, CIBERehd and IDIBAPS; Barcelona, Spain
Marianne Hørby Jørgensen	Rigshospitalet University of Copenhagen; Copenhagen, Denmark
Neil Halliday	Royal Free London NHS Foundation Trust; London, UK
Henning Gronbaek	Aarhus University Hospital, Aarhus, Denmark
Hanns-Ulrich Marschall	Sahlgrenska University Hospital; Gothenburg, Sweden
Romée Snijders	Radboud University Medical Center (UMC); Nijmegen, Netherlands
Ana Lleo	Humanitas University and Humanitas Clinical and Research Center – IRCCS; Rozzano, Milan, Italy
Nora Cazzagon	Azienda Ospedale - Università Padova, Padova, Italy
Natalie Van den Ende	University Hospitals KULeuven; Leuven, Belgium
Maciej K. Janik, Piotr Milkiewicz	Medical University of Warsaw; Warsaw, Poland
Ruben de Kleine	University Medical Center Groningen, Rijksuniversiteit Groningen, Groningen, The Netherlands
Marcin Krawczyk	Saarland University Medical Center; Homburg, Germany
Luigi Muratori	Center for the Study and Treatment of Autoimmune Diseases of the Liver and Biliary System, IRCCS Azienda Ospedaliero-Universitaria di Bologna, Policlinico di S. Orsola; Bologna, Italy
Sandra Ferreira	Centro Hospitalar e Universitário de Coimbra, Portugal
Almaas Runar	Oslo University Hospital, Oslo, Norway
Maria Papp, Boglarka Balogh	Department of Gastroenterology, Faculty of Medicine, Institute of Internal Medicine, University of Debrecen

Table S8. List of all contributing clinicians and centres to COVID-Hep and SECURE-Cirrhosis CLD cohort

List of contributors, with thanks; (submitting clinician, responsible consultant, centre, country)

A S Soin, S K Yadav, Medanta The Medicity, India
Avitabile Emma & Elsa Sola Verges, Hospital Clínic De Barcelona, Spain
Avitabile Emma & Pere Ginãs, Hospital Clínic De Barcelona, Spain
Avitabile Emma, Hospital Universitari Sagrat Cor, Spain
Abigail Ford & Matthew Hoare, Cambridge, UK
Agustina Martinez Garmendia & Ezequiel Mauro, Hospital Italiano De Buenos Aires, Argentina
Ahad Eshraghian & Saman Nikeghbalian, Avicenna Center For Medicine And Organ Transplant, Iran
Ahad Eshraghian & Seyed Ali Malek-Hosseini, Avicenna Center For Medicine And Organ Transplant, Iran
Ahad Eshraghian, Avicenna Center For Medicine And Organ Transplant, Italy
Ahmed Hashim & Jonathan Potts, Royal Free Hospital, UK
Ahmed Hashim & Rachel Westbrook, Royal Free Hospital, UK
Ahmed Hashim & David Patch, Royal Free Hospital, UK
Ahmed Hashim & Jennifer Ryan, Royal Free Hospital, UK
Ahmed Hashim & Aileen Marshall, Royal Free Hospital, UK
Ahmed Hashim & Rosenberg, Royal Free Hospital, UK
Ahmed Hashim & Rajiv Jalan, Royal Free Hospital, UK
Ahmed Hashim & Raj Mokerjee, Royal Free Hospital, UK
Ahmed Tawheed & Mohamed El Kassas, Endemic Medicine Department Helwan University, Egypt
Albert Pares, HCB, Spain
Alessio Gerussi, AMO, Italy
Alvaro Urzua, Hospital Clinico Universidad De Chile, Chile
Amila Mehmedovic, Clinical Centre University Of Sarajevo, Bosnia
Ana Lleo, EEP, Italy
Andrea De Gottardi, Ente Ospedaliero Cantonale, Switzerland
Andrew Moon & Sidney Barritt, University Of North Carolina, USA
Andrew Moon & Scott Elliott, University Of North Carolina, USA
Andrew Moon & Cary Cotton, University Of North Carolina, USA
Andrew Yeoman, Gwent Liver Unit, Royal Gwent Hospital, UK
Ane Soegaard Teisner, Herlev Hospital, Denmark
Anna Crawford & Jane Collier, John Radcliffe Hospital, Oxford., UK

Antonella Putignano & Thierry Gustot, Cub Erasme, Belgium
Apurva Trivedi & Kermit Speeg, University Transplant San Antonio, Tx, USA
Aren & Aren Nersisyan, Mikayelyan Institute Of Surgery, Armenia
Aren Nersisyan, Mikaelyan Institute Of Surgery, Armenia
Aren Nersisyan, Mikaelyan Institute Of Sugery, Armenia
Bilal Bobat, Wits Donald Gordon Medical Centre, South Africa
Blaise Lorraine & Nahon Pierre, Hepatology Unit Jean Verdier Hospital, France
Blaise Lorraine & Grando Véronique , Hepatology Unit Jean Verdier Hospital, France
Blaise Lorraine & Gigante Elia, Hepatology Unit Jean Verdier Hospital, France
Blaise Lorraine & Nault Jean Charles, Hepatology Unit Jean Verdier Hospita, France
Blaise Lorraine & Walter Aurélie , Hepatology Unit Jean Verdier Hospital, France
Blaise Lorraine & Ganne Nathalie, Hepatology Unit Jean Verdier Hospital, France
Blaise Lorraine & Gigante Elia, Hepatology Unit Jean Verdier Hospital, France
Benjamin Mullish & Nowlan Selvapatt, St Mary's Hospital, London, UK
Beth Lusina & Kelly Burak, Foothills Medical Centre, Calgary, Alberta, Canada, Canada
Bethany Robinson & Emilie Wilkes, Nottingham University Hospitals NHS Trust, UK
Bethany Robinson & Neil Guha, Nottingham University Hospitals NHS Trust, UK
Bethany Robinson & M Poon, Nottingham University Hospitals NHS Trust, UK
Bethany Robinson & G Aithal, Nottingham University Hospitals NHS Trust, UK
Bo Wang & Annika Charlesworth, University Hospital Lewisham, UK
Boris Yaremin & Murad Novruzbekov, Sklifosovsky Emergency Medicine Institute, Russia
Bruno Annibale & Massimo Marignani, Ospedale Universitario S. Andrea, Sapienza, Italy
Bulent Baran & Cihan Yurdaydin, Koç University Hospital, Turkey
Carmen Cerron & Martin Padilla, Transplant Department, Peru
Catalina Toledo & Joan Genesca, Vall De Hebron, Spain
Charmaine Matthews & Paul Richardson, Liverpool University Hospitals NHS Foundation Trust, UK
Christina Levick & Richard Aspinall, Queen Alexandra Hospital, UK
Christina Levick & Andrew Fowell, Queen Alexandra Hospital, UK
Colin Smith, Inspira Medical Center Mullica Hill, USA
Costica Aloman & Sujit Janardhan, Rush University Medical Center, USA
Costica Aloman & Nikunj Shah, Rush University Medical Center, USA
Costica Aloman, Rush University Medical Center, USA
Costica Aloman & Justin Mitchell, Rush University Medical Center, USA
Costica Aloman & Sheila Eswaran, Rush University Medical Center, USA
Costica Aloman & Donald Jensen, Rush University Medical Center, USA

Costica Aloman & Nancy Reau, Rush University Medical Centre, USA
Costica Aloman & Justin Mitchel, Rush University Medical Center, USA
Costica Aloman & Nikunj Shaha, Rush University Medical Center, USA
Cristina Fernandez Marcos, Hospital Universitario De Burgos, Spain
Cristina Montana Rodriguez & Martin Prieto, Hospital Universitario La Fe De Valencia, Spain
Cristina Rigamonti, Department of Translational Medicine, Università Del Piemonte Orientale, Italy
Cristina Rigamonti, Division Of Internal Medicine, Azienda Ospedaliero Universitaria Maggiore Della Carità Di Novara, Italy
Daniel Ochoche Amedu, Frsc Medical Centre Gwarimpa, Nigeria
Daniela Hernández Castro, Hospital México , Costa Rica
Daniele Nicolini & Marco Vivarelli, Department Of Gastroenterology And Transplantation, Polytechnic University Of Marche, Ancona, Italy, Italy
Daniele Nicolini & Marco Vivarelli, Division Of Hepatobiliary, Pancreatic And Abdominal Transplant Surgery, A.O.U. Ospedali Riuniti Di Ancona, Italy
David Harman & Jane Collier, Oxford University Hospitals, UK
David Wong, Toronto General Hospital, Canada
David Wong, University Of Toronto, Canada
Debbie Shawcross & Michael Heneghan, King's College Hospital, UK
Debbie Shawcross & Aluvihare/Suddle/Shawcross/Heneghan, King's College Hospital, UK
Devina Bhasin, Piedmont Transplant Institute, USA
Austin, Royal Derby Hospital, UK
Ben Hudson, Royal Devon and Exeter NHS Foundation Trust, UK
Emily Glynn & John Ryan, Beaumont Hospital, Ireland
Heather Lafferty & Kenneth Simpson, Scottish Liver Transplant Unit, Edinburgh Royal Infirmary, UK
Hrishikesh Samant, Ochsner Louisiana State University Health, Shreveport, USA
Kate Axe & Sekina Ghuman, Gwent Liver Unit, UK
Manas, All India Institute of Medical Sciences, India
Manas Vaishnav & Shalimar, All India Institute of Medical Sciences, India
Matthew Armstrong & Ferguson, Queen Elizabeth Hospital, Birmingham, UK
Matthew Armstrong & Oo, Queen Elizabeth Hospital, Birmingham, UK
Mhairi C Donnelly & Mhairi C Donnelly, Freeman Hospital, Newcastle, Uk, UK
Roberta Elisa Rossi & Vincenzo Mazzaferro, Istituto Nazionale Dei Tumori, Milan, Italy
Roger Mccorry & Neil Mcdougall, Royal Victoria Hospital, Belfast, UK
Rooshi Nathwani & Ameet Dhar, St Mary's Hospital, UK
Sherrie Bhoori & Vincenzo Mazzaferro, Istituto Nazionale Dei Tumori, Milan, Italy
Upkar Gill & Vikram Sharma, Royal London Hospital (Barts Health NHS Trust), UK
Upkar Gill & Paul Kooner, Royal London Hospital (Barts Health NHS Trust), UK
Upkar Gill & Yiannis Kallis, Royal London Hospital (Barts Health NHS Trust), UK

Upkar Gill & William Alazawi, Royal London Hospital (Barts Health NHS Trust), UK
Upkar Gill & Sudeep Tanwar, Whipps Cross University Hospital (Barts Health NHS Trust), UK
Upkar Gill & Patrick Kennedy, Royal London Hospital (Barts Health NHS Trust), UK
Upkar Gill & Graham Foster, Royal London Hospital (Barts Health NHS Trust), UK
Upkar Gill & Richard Marley, Royal London Hospital (Barts Health NHS Trust), UK
Upkar Gill & Sushma Saksena, Royal London Hospital (Barts Health NHS Trust), UK
Upkar Gill & Vikram Sharma, Newham University Hospital (Barts Health NHS Trust), UK
Upkar Gill & Graham Foster, Whipps Cross University Hospital (Barts Health NHS Trust), UK
Upkar Gill & Aruna Dias, Newham University Hospital (Barts Health NHS Trust), UK
Upkar Gill & Janet Dearden, Whipps Cross University Hospital (Barts Health NHS Trust), UK
Hinrichsen & Rainer Guenther, Department Of Internal Medicine/Liver Unit, Germany
Sarang Thaker & Adam Mikolajczyk, University Of Illinois Hospital, USA
Emma Avitabile & Zoe Mariã, Hospital Clinic Barcelona, Spain
Emma Avitabile & Elsa Sola Verges, Hospital Clinic Barcelona, Spain
Emma Avitabile & Elisa Pose Mendez, Hospital Clinic De Barcelona, Spain
Emma Avitabile & Isabel Graupera Garcia, Hospital Clinic De Barcelona, Spain
Emma Avitabile & Pere Gines, Hospital Clinic De Barcelona, Spain
Emma Avitabile & Albert Pares, Hospital Clinic De Barcelona, Spain
Emma Avitabile & Juan Carlos Garcia Pagan, Hospital Clinic Barcelona, Spain
Emma Avitabile & Marco Sanduzzi Zamparelli, Hospital Clinic De Barcelona, Spain
Emma Avitabile & Maria Martinez Rebollar, Hospital Clinic De Barcelona, Spain
Emma Avitabile & Cristina Sole Marti, Hospital Clinic De Barcelona, Spain
Emma Avitabile & Alejandro Forner Gonzalez, Hospital Clinic Barcelona, Spain
Emma Avitabile & Xavier Forns Bernhardt, Hospital Clinic De Barcelona, Spain
Emma Avitabile & Maria Carlota Londoã, Hospital Clinic De Barcelona, Spain
Emma Avitabile & Isabel Graupera, Hospital Clinic Barcelona, Spain
Eabha Ring & Steve Stewart, Mater University Hospital Dublin, Ireland
Eduardo Rodriguez, University Of Utah, USA
Elton Dajti & Mariarosa Tamã¨, Sant'Orsola Hospital, Italy
Esteban Fuentes Valenzuela & Julia Gomez Barquero, Hospital Universitario Rio Hortega, Spain
Ewan Forrest, Glasgow Royal Infirmary, UK
Felipe Alconchel & Pablo RamãRez, Virgen De La Arrixaca University Hospital, Spain
Feng Su, Harborview Medical Center, USA
Feng Su & Michele Goodman Arnp, University Of Washington Northwest Hospital, USA
Feng Su & Charles Landis, Harborview Medical Center, USA

Feng Su & Iris Liou, University Of Washington, USA
Feng Su & Paula Cox-North Arnp, Harborview Medical Center, USA
Filipa Bordalo Ferreira & Maria Ana Rafael, Hospital Professor Doutor Fernando Fonseca, Portugal
Filipa Bordalo Ferreira & Alexandra Martins, Hospital Professor Doutor Fernando Fonseca, Portugal
Filipa Bordalo Ferreira & Joana Milheiro Branco, Hospital Professor Doutor Fernando Fonseca, Portugal
Filipa Bordalo Ferreira & Eugénio Teófilo, Hospital De Santo António Dos Capuchos , Portugal
Filipa Bordalo Ferreira & Maria Luísa Figueiredo, Hospital Professor Doutor Fernando Fonseca, Portugal
Filpe Calinas & Filipe Calinas, Centro Hospitalar Lisboa Central, Portugal
Francesca Saffioti, Oxford University Hospitals NHS Foundation Trust, UK
Francesca Saffioti & Jeremy Cobbold, Oxford Liver Unit, Oxford University Hospitals, UK
Gabriel Aballay Soteras, Instituto De Trasplante Y Alta Complejidad, Argentina
Gupse Adali, University Of Health Sciences, Umraniye Training And Research Hospital, Istanbul, Turkey, Turkey
Gupse Adali & Sbu Umraniye Training And Research Hospital, Sbu Umraniye Training And Research Hospital, Turkey
Gabriel Aballay Soteras, Argerich Hospital, Argentina
Gabriel Aballay Soteras, Instituto De Trasplantes Y Alta Complejidad,
Gloria Torres & Joan Genescà, Hospital Vall D'Hebron, Spain
Graham Brady & Robert Fontana, University Of Michigan, USA
Gustav Buescher & Christoph Schramm, University Medical Center Hamburg, Germany
Gustavo Henrique Santos Pereira, Hepatology and Liver Transplantation Division, Brazil
Gwilym Webb & Jane Collier, Oxford University Hospitals, UK
Hannes Hagström & Annika Bergquist, Karolinska University Hospital, Sweden
Hannes Hagström & Joel Marmur, Karolinska University Hospital, Sweden
Hannes Hagström & Staffan Wahlin, Karolinska University Hospital, Sweden
Hannes Hagström & Ammar Barakat, Karolinska University Hospital, Sweden
Hannes Hagström & Lina Lindström, Karolinska University Hospital, Sweden
Hannes Hagström & Mattias Lissing, Karolinska University Hospital, Sweden
Hannes Hagström & Antti Oksanen, Karolinska University Hospital, Sweden
Hannes Hagström & Per Stål, Karolinska University Hospital, Sweden
Hannes Hagström & A.Romano, Karolinska University Hospital, Sweden
Hanns-Ulrich Marschall, SUH, Sweden
Heather Javaid & Jagadish Nagaraj, Morriston Hospital, Swansea, UK
Heather Lafferty & Adrian Stanley, Glasgow Royal Infirmary, UK
Henning Groenbaek, AUH, Denmark
Iain Ewing, Homerton University Hospital, UK
Ignacio García Juárez, El Instituto Nacional De Ciencias Médicas Y Nutrición Salvador Zubirán, Mexico

Ignacio García Juárez & Francisco I García-Juárez, Regional Hospital Lic. Adolfo Lopez Mateos Issste, Mexico
Ignacio García Juárez & José A Avila Rojo , El Instituto Nacional De Ciencias Médicas Y Nutrición Salvador Zubirán, Mexico
Ignacio García Juárez, El Instituto Nacional De Ciencias Médicas Y Nutrición Salvador Zubirán,
Ignacio García Juárez & Luis A Estrella Sato, El Instituto Nacional De Ciencias Médicas Y Nutrición Salvador Zubirán, Mexico
Ignacio García Juárez & Alan G Contreras, El Instituto Nacional De Ciencias Médicas Y Nutrición Salvador Zubirán, Mexico
Ignacio García Juárez & Jesus A Camacho Escobedo, Hospital Almater, Mexicali, Mexico, Mexico
Isaac Ruiz & Geneviève Huard, Centre Hospitalier De L'Université De Montréal (CHUM), Canada
Isaac Ruiz & Hélène Castel, Centre Hospitalier De L'Université De Montréal (CHUM), Canada
Isaac Ruiz & Claire Fournier, Centre Hospitalier De L'Université De Montréal (CHUM),
Isaac Ruiz & Julien Bissonnette, Centre Hospitalier De L'Université De Montréal (CHUM),
Isaac Ruiz & Jean-Pierre Villeneuve, Centre Hospitalier De L'Université De Montréal (CHUM), Canada
Isaac Ruiz & Jeanne-Marie Giard, Centre Hospitalier De L'Université De Montréal (CHUM), Canada
Isaac Ruiz & Catherine Vincent, Centre Hospitalier De L'Université De Montréal (CHUM), Canada
Isaac Ruiz & Marc Bilodeau, Centre Hospitalier De L'Université De Montréal (CHUM), Canada
Isaac Ruiz & Raymond Leduc, Centre Hospitalier De L'Université De Montréal (CHUM), Canada
Judith Gomez Camarero & Cristina Fernandez Marcos, Hospital Universitario De Burgos, Spain
James Esteban & Acuthan Sourianarayanane, Froedtert and Medical College Of Wisconsin, USA
James Esteban & Kia Saeian, Froedtert and The Medical College Of Wisconsin, USA
James Esteban & Syed Rizvi, Froedtert and Medical College Of Wisconsin, USA
James Esteban & Aiman Ghufran, Froedtert and Medical College Of Wisconsin, USA
James Esteban & Kia Saeian, Froedtert and Medical College Of Wisconsin, USA
Janisha Patel, University Hospital Southampton NHSFT, UK
Jennifer Ray & Alexa Fider-Whyte, Saint Louis University Hospital, USA
Jihane Benhammou, UCLA, USA
Johnny Cash & Ian Cadden, Royal Victoria Hospital Belfast, UK
Jonathan Crisostomo & Arlinking Ong-Go, Metropolitan Medical Center, Philippines
Joost PH Drenth & Eric Tjwa, Erasmusmc Rotterdam, Netherlands, The Netherlands
Joost PH Drenth, UMCG, Groningen Netherlands, The Netherlands
José Presa, Liver Unit-Chtmad, Portugal
Juan Pablo Arab Verdugo, Hospital Clinico Universidad Catolica De Chile, Chile
Judith Gomez Camarero & Belen Bernad Cabredo, Hospital Universitario De Burgos, Spain
Juozas Kupcinskas, Lithuanian University Of Health Sciences, Lithuania
Justin Boike & Laura Kulik, Northwestern, USA
Justin Boike & Josh Levitsky, Northwestern, USA
Justin Boike & Daniel Ganger, Northwestern, USA

Justin Boike & Christopher Moore, Northwestern, USA
Justin Chin & Varuna Aluvihare, Kings College Hospital, UK
Justin Chin & Vishal Patel, Kings College Hospital, UK
Justin Chin & Kosh Agarwal, Kings College Hospital, UK
Konstantina Nikitopoulou & Andrew Johnston, Cambridge University Hospitals NHS Foundation Trust, UK
Konstantina Nikitopoulou & Keval Naik, Cambridge University Hospitals NHS Foundation Trust, UK
Konstantina Nikitopoulou & Michael Allison, Cambridge University Hospitals NHS Foundation Trust, UK
Konstantina Nikitopoulou & Victoria Snowdon, Cambridge University Hospitals NHS Foundation Trust, UK
Konstantina Nikitopoulou & George Mells, Cambridge University Hospitals NHS Foundation Trust, UK
Konstantina Nikitopoulou & Joanna Leithead, Cambridge University Hospitals NHS Foundation Trust, UK
Konstantina Nikitopoulou & William Griffiths, Cambridge University Hospitals NHS Foundation Trust, UK
Konstantina Nikitopoulou & Raaj Praseedom, Cambridge University Hospitals NHS Foundation Trust, UK
Konstantina Nikitopoulou & Paul Gibbs, Cambridge University Hospitals NHS Foundation Trust, UK
Konstantina Nikitopoulou & Michalis Kostapanos, Cambridge University Hospitals NHS Foundation Trust, UK
Konstantina Nikitopoulou & Matthew Hoare, Cambridge University Hospitals NHS Foundation Trust, UK
Katherine Marx & Maria Andreea Catana, Beth Israel Deaconess Medical Center, USA
Kevin Korenblat, Washington University School Of Medicine, USA
Kiersten Berg & Alex Aspinall, Southern Alberta Transplant Program, Canada
Kiersten Berg & Stephen Congly, Foothills Medical Center, Canada
Kuldeep Cheent, Frimley Park Hospital, UK
Lance L Stein, Piedmont Atlanta Hospital, USA
Leanne Stratton, Royal Victoria Hospital, Belfast, UK
Logan Hobbs & Craig Lammert, IU Health University Hospital, USA
Logan Hobbs & Marwan Ghabril, IU Health University Hospital, USA
Logan Hobbs & Archita Desai, IU Health University Hospital, USA
Luigi Muratori, POM, Italy
Maria Fernanda Guerra Veloz, Hospital Universitario Virgen Macarena, Spain
Maria Fernanda Guerra Veloz & Maria Jose Rios, Hospital Universitario Virgen Macarena, Spain
Maria Fernanda Guerra Veloz & Patricia Cordero Ruiz, Hospital Universitario Virgen Macarena, Spain
Maciej K. Janik & Piotr Milkiewicz, Medical University Of Warsaw, Poland, Poland
Marcella Salzano & Joan Genesca, Hospital Vall D'Hebron, Spain
Marcin Krawczyk, SUM, Germany
Marco Distefano, UOC Malattie Infettive Asp8, Italy
Maria Andreea Catana & Alan Bonder, Beth Israel Deaconess Medical Center, USA
Maria Andreea Catana & Kathleen Corey, MGH, USA

Maria Andreea Catana & Nezam Afdhal, Beth Israel Deaconess Medical Center, USA
Maria Andreea Catana, Beth Israel Deaconess Medical Center, USA
Maria Andreea Catana & Patwardhan, Vilas, Beth Israel Deaconess Medical Center, USA
Maria Andreea Catana & Michelle Lai, Beth Israel Deaconess Medical Center, USA
Maria Andreea Catana & Micheal Curry, Beth Israel Deaconess Medical Center, USA
Maria Andreea Catana & Vilas Patwardhan, Beth Israel Deaconess Medical Center, USA
Maria Andreea Catana & Lau, Daryl, Beth Israel Deaconess Medical Center, USA
Maria Andreea Catana & Raza Malik, Beth Israel Deaconess Medical Center, USA
Maria Andreea Catana Md & Fricker, Zachary P. Beth Israel Deaconess Medical Center, USA
Maria Andreea Catana Md & Afdhal, Nezam H. Beth Israel Deaconess Medical Center, USA
Maria Londono, HCB, Spain
Maria Torrens & Joan Genescã , Hospital Vall Dâ´Hebron, Spain
Maria-Andreea Catana & Tara Ghaziani, Beth Israel Deaconess Medical Center, USA
Marianne Hoerby-Joergensen, RUC, Denmark
Mariarosa Tamã, Sant'Orsola Hospital, Bologna, Italy
Mark Sonderup, University Of Cape Town, South Africa
Martin Prince, Manchester Royal Infirmary, UK
Mary Drinane, Dartmouth-Hitchcock Medical Center, USA
Mary Kouba & Raymond Rubin, Piedmont Atlanta Hospital, USA
Mary Kouba & Devina Bhasin, Piedmont Atlanta Hospital, USA
Massimo Iavarone, Foundation IRCCS Ca' Granda Ospedale Maggiore Policlinico, Milan, Italy
Matias Estevez Escobar & Cristina Vinolo Ubina, Hospital De Poniente, Spain
Matias Estevez Escobar, Hospital De Poniente, Spain
Matthew Armstrong & Mutimer, Queen Elizabeth Hospital, Birmingham, UK
Matthew Armstrong & Tripathi, Queen Elizabeth Birmingham, Birmingham, UK
Matthew Armstrong & Thompson, Queen Elizabeth Hospital, Birmingham, UK
Matthew Armstrong & Thamara, Queen Elizabeth Hospital, Birmingham, UK
Matthew Foxton, Chelsea & Westminster Hospital, UK
Matthew Mcconnell & Cary Caldwell, Yale University, USA
MaRa Godoy, Santa Isabel Hospital, Brazil
Mhairi Donnelly & Mark Hudson, Freeman Hospital, Newcastle, UK
Michael Andrew Yu & Ram Subramanian, Emory Transplant Center, USA
Michael J Monzel, Digestive Disease Center Of The Palm Beaches, USA
Milena Mendes, Unidade De Transplante- Hospital Curry Cabral, Portugal
Ming-Hua Zheng, The First Affiliated Hospital Of Wenzhou Medical University, China

Mohamed Elfeki, Iowa Methodist Medical Center, USA
Mohamed Elfeki & Donald Hillebrand, Iowa Methodist Medical Center, USA
Mohamed Elfeki & Jason Kruse, Broadlawns Medical Center, USA
Monica Cucco & Gianluca Svegliati Baroni, Department Of Gastroenterology, Polytechnic University Of Marche, Ancona, Italy, Italy
Nikolaos Gatselis, G.N. Dalekos) & Sp. Potamianos, University Hospital Of Larissa, Greece, Greece
Nikolaos Gatselis, G.N. Dalekos & E. Akriviadis, Diavalkaniko Center, Thessaloniki, Greece, Greece
Nancy Reau, Rush, USA
Natalie Van Den Ende, UHL, Belgium
Neil Shah, UNC Hospitals, USA
Nicola Pugliese, Uo Medicina Interna Ed Epatologia, Dipartimento Di Gastroenterologia, Italy
Nneka Ufere & Karim Fawaz, Somerville Hospital, USA
Nneka Ufere & Michael Thiim, Massachusetts General Hospital, USA
Nneka Ufere & Shoko Kimura, Massachusetts General Hospital, USA
Nneka Ufere & Gary Trey, Cambridge Health Alliance, USA
Nneka Ufere & Lizabeth Cline, Boston Medical Center, USA
Nneka Ufere & Panagiotis Trilianos, Boston Medical Center, USA
Nneka Ufere & Kathleen Corey, Massachusetts General Hospital, USA
Nneka Ufere & Tracey Simon, Massachusetts General Hospital, USA
Nneka Ufere & Connie Huang, North Shore Medical Center, USA
Nneka Ufere & Stephen Zucker, Brigham and Women'S Hospital, USA
Nora Cazzagon, OUP, Italy
Nuru Bayramov, Azerbaijan Medical University, Azerbaijan
Nurun Tania & Abouda, Hull University Teaching Hospitals, UK
Nurun Tania & George Abouda, Hull University Teaching Hospitals, UK
Dominik Bettinger & Robert Thimme, University Medical Center Freiburg, Germany
Patricia D Jones & Martin, University Of Miami, USA
Patricia D. Jones & O'Brien, University Of Miami, USA
Patricia D. Jones & Arosemena, University Of Miami, USA
Patricia Jones & Shane, University Of Miami, USA
Patricia Jones & Molliner, University Of Miami, USA
Pedro Montes, Hospital Nacional Daniel Alcides Carrion, Peru
Ponni Perumalswami & Kamron Pourmand, Mount Sinai Hospital, USA
Ponni Perumalswami & Amon Ashgarpour, Mount Sinai Hospital, USA
Ponni Perumalswami & Ritu Agarwal, Mount Sinai Hospital, USA
Ponni Perumalswami & Amon Asgharpour, Mount Sinai Hospital, USA

Ponni Perumalswami & Thomas Schiano, Mount Sinai Hospital, USA
Ponni Perumalswami & Leona Kim-Schluger, Mount Sinai Hospital, USA
Ponni Perumalswami & Charissa Chang, Mount Sinai Hospital, USA
Ponni Perumalswami & Gene Im, Mount Sinai Hospital, USA
Ponni Perumalswami & Linda Law, Mount Sinai Hospital, USA
Ponni Perumalswami & Jawad Ahmad, Mount Sinai Hospital, USA
Ponni Perumalswami & Jennifer Leong, Mount Sinai Hospital, USA
Ponni Perumalswami & Joseph Odin, Mount Sinai Hospital, USA
Ponni Perumalswami & James Crismale, Mount Sinai Hospital, USA
Ponni Perumalswami & Priya Grewal, Mount Sinai Hospital, USA
Qi Xiaolong & Chess, Chess, China
Rafael Oliveira Ximenes & Aline De Castro Pereira, Hospital Do Coração De Goiás, Brazil
Rahul Maheshwari, Piedmont Hospital, USA
Rajiv Majithia, Rex Digestive Healthcare, USA
Raymond Rubin, Piedmont Atlanta Hospital, USA
Raymond Rubin & Rahul Maheshwari, Piedmont Atlanta Hospital, USA
Richard Parker, Leeds Teaching Hospitals NHS Trust, UK
Romee Snijders, UMR, The Netherlands
Rooshi Nathwani & Pinelopi Manousou, St Mary's Hospital, Paddington, UK
Rooshi Nathwani & Lucia Possamai, St Mary's Hospital, Paddington, UK
Rooshi Nathwani & Ameet Dhar, St Mary's Hospital, Paddington, UK
Rooshi Nathwani & William Howson, Charing Cross Hospital, UK
Rooshi Nathwani & Heather Lewis, St Mary's Hospital, Paddington, UK
Rooshi Nathwani & Ameet Dhar, St Mary's Hospital, UK
Rooshi Nathwani & Nowlan Selvapatt, St Mary's Hospital, Paddington, UK
Sarah Townsend & Jane Collier, John Radcliffe Hospital, UK
Sarah Townsend & Douglas Thorburn, Royal Free Hospital, UK
Sarang Thaker & Sean Koppe, University Of Illinois Hospital, USA
Sarang Thaker & Adam Mikolajczyk, University Of Illinois Hospital, USA
Sathish Subramanian, MGH, USA
Sekina Ghuman, Gwent Liver Unit, UK
Sherief Abd-Elsalam, Tanta University, Egypt
Soek-Siam Tan, Selayang Hospital, Malaysia
Sonia Blanco Sampascual & Blanco Sampascual, Hospital Universitario Basurto, Spain
Sonia Blanco Sampascual & Menendez, Hospital Universitario Basurto, Spain

Sonia Blanco Sampascual & Castro, Hospital Universitario Basurto, Spain
Stephen Barclay, Glasgow Royal Infirmary, UK
Stephen Barclay & Ewan Forrest, Glasgow Royal Infirmary, UK
Steven Masson & Louise Macdougall, Newcastle Hospitals, UK
Steven Masson & Mark Hudson, Newcastle Hospitals, UK
Steven Masson & Donnelly, Newcastle Hospitals, UK
Tanvir Haque, UNC Rex Hospital, USA
Teresa Broquetas, Hospital Del Mar (Barcelona), Spain
Thines Karunakaran & Chirag Oza, Broomfield Hospital, UK
Thinesh Lee Krishnamoorthy, Singapore General Hospital, Singapore
Thinesh Lee Krishnamoorthy & Clement Wu, Singapore General Hospital, Singapore
Thomas Marjot, Oxford University Hospitals NHS Trust, UK
Ulrich Thalheimer, Royal Shrewsbury Hospital, UK
Valérie Mclin, Swiss Pediatric Liver Center, Switzerland
Vanesa Bernal, Hospital Universitario Miguel Servet, Spain
Vanessa De Villa, The Medical City, Philippines
Veronica Nguyen & Geoffrey Block, Banner University Medical Center/University Of Arizona, Tucson, USA
Vincent Cheung & James Maggs, Stoke Mandeville Hospital, UK
Wim Laleman, University Hospital Leuven, Belgium
Wong Yu Jun, Changi General Hospital, Singapore
Xavier Verhelst, Ghent University Hospital Belgium, Belgium
Xiaolong Qi & CHESS, Guangdong, China
Xiaolong Qi & CHESS, Hubei, China
Xiaolong Qi & CHESS, Jiangsu, China
Xiaolong Qi & CHESS, Guangxi, China
Xiaolong Qi & CHESS, Tianjin, China
Zurabi Lominadze, University Of Maryland Medical Center, USA

Fig. S1. Non-CLD cohort selection

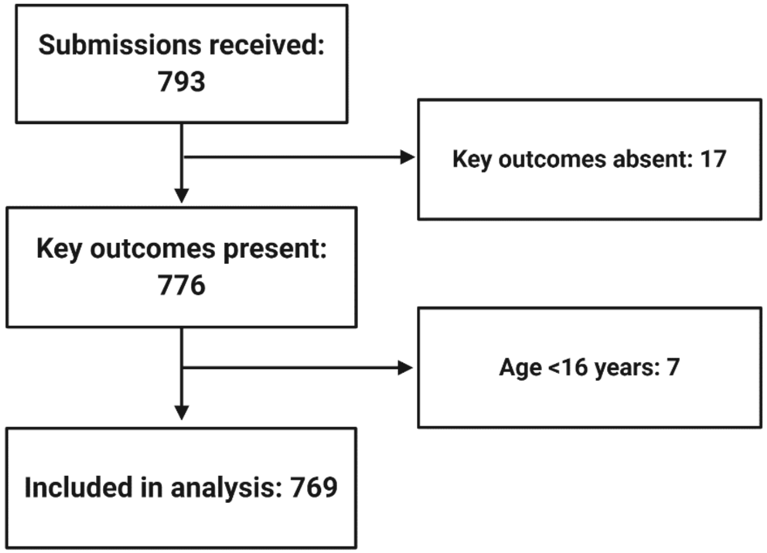

Supplementary figure 1. Total combined consecutive cases of patients with SARS-CoV-2 infection without liver disease reported to Oxford University Hospitals NHS Trust and University of North Carolina Hospitals during study period and number included in the analysis after exclusions.
