# Supplementary material for: SARS-CoV-2 infection in patients with autoimmune hepatitis
Source: J Hepatol. 2021 Jun;74(6):1335–43. doi: 10.1016/j.jhep.2021.01.021 (PMC7835076; doi:10.1016/j.jhep.2021.01.021)
Supplement: Multimedia component 4 [file mmc4.pdf]

JOURNAL
OF HEPATOLOGY

Journal of Hepatology

CTAT methods

Tables for a "Complete, Transparent, Accurate and Timely account" (CTAT) are now mandatory for all revised submissions. The aim is to enhance the reproducibility of methods.

- Only include the parts relevant to your study
- Refer to the CTAT in the main text as 'Supplementary CTAT Table'
- Do not add subheadings
- Add as many rows as needed to include all information
- Only include one item per row

If the CTAT form is not relevant to your study, please outline the reasons why:

None of the domains described below are relevant to this this international registry study of COVID-19 in patients with autoimmune hepatitis.

1.1 Antibodies

Name	Citation	Supplier	Cat no.	Clone no.

1.2 Cell lines

Name	Citation	Supplier	Cat no.	Passage no.	Authentication test method

1.3 Organisms

Name	Citation	Supplier	Strain	Sex	Age	Overall n number

1.4 Sequence based reagents

Name	Sequence	Supplier

1.5 Biological samples

Description	Source	Identifier

1.6 Deposited data

Name of repository	Identifier	Link

1.7 Software

Software name	Manufacturer	Version

1.8 Other (e.g. drugs, proteins, vectors etc.)

1.9 Please provide the details of the corresponding methods author for the manuscript:

Prof. Ansgar W. Lohse
Department of Medicine, University Medical Centre Hamburg-Eppendorf
Martinistr. 52, D-20246, Hamburg, Germany
Tel.: +49(0)40 7410 53910
Email : alohse@uke.de

2.0 Please confirm for randomised controlled trials all versions of the clinical protocol are included in the submission. These will be published online as supplementary information.

